# A real-time mechanistic framework for early inference of chikungunya transmission and outbreak sizes in mainland France, 2025

**DOI:** 10.1371/journal.pntd.0014534

**Published:** 2026-07-13

**Authors:** Sandeep Tegar, Dominic P. Brass, Bethan V. Purse, Antoine Mignotte, Guillaume Lacour, Christina A. Cobbold, Steven M. White

**Affiliations:** 1 UK Centre for Ecology & Hydrology, Benson Lane, Wallingford, Oxfordshire, United Kingdom; 2 School of Mathematics and Statistics, College of Science and Engineering, University of Glasgow, Glasgow, United Kingdom; 3 Altopictus, Pérols, France; George Washington University Medical Center, UNITED STATES OF AMERICA

## Abstract

In 2025, mainland France experienced an unprecedented chikungunya outbreak in Europe, likely associated with suitable climatic conditions and established presence of *Aedes albopictus*, the primary European vector of chikungunya virus (CHIKV). During the early phase of the outbreak (May-July 2025), when only limited case data were available, public health decision-making required rapid situational assessment under substantial uncertainty. Here, we apply a state-of-art, climate-sensitive eco-epidemiological modelling framework to infer transmission dynamics in real-time and to characterise early progression of outbreaks across affected locations in France. The framework is designed to infer transmission relevant information from early epidemic data, including relative outbreak risk across locations, potential timing of the initial amplification phase of transmission, and likely size of outbreaks in the absence of public-health control interventions. The analysis indicates that early epidemic data can be used to identify locations likely to experience substantial transmission episodes and to refining estimates of the timing of key transition phases of transmission, including the initial amplification phase of transmission approximately 2–3-months ahead of peak transmission. Predicted outbreak potential varied considerably across locations, reflecting differences in local climatic conditions and population density incorporated within the modelling framework. This framework supports real-time situational awareness and can be employed to improve the response and targeting of public health interventions during the emerging phase of outbreaks. Furthermore, insights into location-specific differences in predicted outbreak size and response priorities are particularly useful for understanding the localised transmission risk and informing targeted interventions.

## Author summary

Mainland France experienced an unprecedented chikungunya outbreak in 2025, representing the largest locally transmitted (autochthonous) outbreak in mainland Europe at the national scale. Between 2010, when the first two autochthonous cases were reported, and 2024, only 32 autochthonous cases had been documented. In contrast, the 2025 outbreak, resulted in 809 autochthonous cases in France, highlighting the rapidly increasing scale of chikungunya transmission in Europe.

During the early phase of the France outbreak (May-July 2025), when only limited case data were available, substantial uncertainty existed at the level of individual locations regarding the potential transmission and the timing of interventions.

In this study, we apply a climate-sensitive mechanistic modelling framework to infer early transmission phase of chikungunya virus (CHIKV) and to characterise how climate variability and vector ecology can shape the epidemic progression.

This framework infers differences in localised transmission potential and provides early information on location-specific relative potentials, such as total case numbers and timings of peak cases, with 2–3 months of lead time before peak transmission.

Results of this study demonstrate how mechanistic models can support real-time assessment of outbreak risk and decision support during the early stages of CHIKV transmission by *Ae. albopictus*, when timely public health interventions are more responsive.

## Introduction

Chikungunya virus (CHIKV) is a debilitating *Aedes*-borne alphavirus that is typically endemic in tropical climates [1]. Since 2007, episodes of locally transmitted (autochthonous) cases of CHIKV have been repeatedly detected in Europe, which are directly linked to the expanding presence of *Aedes albopictus* (Asian tiger mosquito), the primary European vector of CHIKV [2]. Recently, in 2025, the established population of *Ae. albopictus* combined with suitable climatic conditions contributed to an unprecedented CHIKV outbreak, with 809 autochthonous cases reported across 79 locations in mainland France [3]. In contrast, between, 2010 and 2024, only a small number of autochthonous cases were reported in the country. Before 2024, these outbreaks in France occurred in only two or three locations in a year, usually involved few cases, and were generally initiated by travellers returning from endemic regions [1,2].

In mainland France, routine epidemiological surveillance plays a central role in public‑health efforts to detect, prevent, and control outbreaks. Surveillance has been conducted since 2006 [4], with intensified monitoring during the period when *Ae. albopictus* is active (1^st^ May through 30^th^ November), and it requires the systematic notification of probable (suspected) and confirmed cases [5–7]. The system mandates that clinical laboratories immediately report both imported and locally acquired chikungunya cases to Santé Publique France (SPF) and the Regional Health Agencies (Agences régionales de santé, ARS) [4]. The ARS then identifies the relevant physical locations and assesses the intensity and urgency of implementing preventive and vector‑control measures. Overall, surveillance aims to ensure the timely identification of human cases and to implement public‑health and vector‑control strategies to limit the risk of autochthonous transmission [3,5].

However, the effectiveness of routinely employed surveillance is often constrained by uncertainties related to underreporting, reporting delays, and the difficulty of identifying the index case, which can allow the pathogen to spread geographically before detection and the initiation of intervention plans. For instance, during the 2025 chikungunya outbreak in France, a substantial proportion of imported cases (666 of 833 reported by the end of July 2025) originated from the large concurrent epidemic on La Réunion, with additional importations linked to other locations such as Mauritius, Madagascar, Mayotte and Sri Lanka [8]. Although these importations were recognised through routine surveillance, real‑time detection and confirmation of individual infections is inherently limited for emerging pathogens, contributing to uncertainty around the timing and location of initial introductions. These challenges are further compounded by the strong environmental‑sensitivity of the vector‑pathogen system (e.g., [9,10]). Previous studies have identified delayed reporting of autochthonous cases as a key driver of outbreak amplification, with average reporting delays ranging from approximately 9 days to more than 21 days [6,11].

Moreover, a recent assessment of surveillance and control performance during CHIKV outbreaks in Bergerac, France, highlights that delays in implementing response measures after case detection can also substantially reduce their effectiveness, particularly because environmental variation across locations can allow transmission chains to progress before interventions take effect [12].

Mechanistic modelling can be employed to address many of the real‑time challenges and uncertainties faced by surveillance and control programmes. However, mechanistic models have traditionally been used to hindcast past outbreaks and to generate long‑range projections under different climatic or environmental scenarios (e.g., [13–20]), and have rarely been applied to support real‑time inference during emerging vector‑borne diseases (e.g., [12,21]). Validated and tractable mechanistic models can be applied in real-time to infer unobserved states and to integrate climatic fluctuations, temperature‑sensitive development delays, and vector–pathogen dynamics, all of which are essential for understanding how transmission chains unfold in real time (e.g., [9,12,22]). Integrating surveillance programmes with real‑time mechanistic inference tailored to a specific vector–pathogen system is essential for strengthening outbreak‑risk assessment and supporting more timely and effective control decisions [7,23].

In this study, we apply a state-of-the-art climate-sensitive eco-epidemiological framework tailored to the CHIKV-*Ae. albopictus* system to infer transmission dynamics in real time and to characterise the early progression of the chikungunya outbreak notified in mainland France during its initial phase (May-July 2025). This framework uses location-specific case data from the early phase of the epidemic to estimate relative outbreak risk across locations, identify the likely onset of the initial amplification phase of transmission, and assess the potential size of emerging outbreaks.

## Methods

### Ethics statement

The analyses conducted in this study were based on aggregated data without personal identifiers, consequently ethical review was not required

### Modelling the real-time *Ae. albopictus* abundance and CHIKV transmission dynamics

We adopted an extensively validated mechanistic framework for dengue transmission by *Ae. albopictus*, which incorporates climate-sensitive epidemiological traits, stage-structured mosquito population dynamics, and temperature-dependent development delays [9,24]. Although originally developed for dengue, the framework can be extended to chikungunya transmission by *Ae. albopictus* provided that climate-sensitive epidemiological traits for the relevant vector-pathogen system are available. To achieve this, we modelled the thermal performance curves for the extrinsic incubation period (EIP; the time between an adult female mosquito feeding on an infected human and becoming infectious) and vector competence of the CHIKV–*Ae. albopictus* system [10] (Table A in S1 Text), re‑parameterised the original *Ae. albopictus*–dengue framework [9] for chikungunya transmission and verified that the resulting framework reproduced epidemiological features of historical chikungunya outbreaks in Europe (e.g., Bergerac [12]).

The reparameterised state-of-the-art dynamic model characterises CHIKV transmission using a Susceptible–Exposed–Infected–Recovered (SEIR) epidemic framework integrated with the environmentally driven, stage‑ and phenotype‑structured population and trait dynamics of *Ae. albopictus* (Fig A in S1 Text). These environmentally driven trait dynamics capture the phenotypic plasticity of the invasive mosquito *Ae. albopictus* in novel environments, enabling real‑time representation of eco‑epidemiological traits relevant to mosquito‑borne disease transmission, including mosquito longevity, egg diapause and quiescence, development rate, stage‑specific survival probabilities, biting rate, and the virus’s extrinsic incubation period (detailed in [9]).

In addition, the modelling framework (e.g., [9]) has been extensively validated against *Ae. albopictus* life‑history traits, global patterns of vector population seasonality, and European dengue outbreaks, including the more recent Fano and Lodi outbreaks in Italy [22]. In each validation, the model predicted the dengue cases and seasonal dynamics of *Ae. albopictus* across all the life‑stages with high accuracy at different times across different locations globally. These evaluations demonstrate that the framework reliably captures both vector-population dynamics and pathogen-transmission dynamics, supporting its transferability to the *Ae. albopictus*–CHIKV system for real‑time inference.

### French CHIKV case data (2025)

Case data used in this study were gathered from published reports by Santé Publique France (SPF) and the Agences Régionales de Santé (ARS) [8]. Here, we only use data from the early transmission period (1 May to 22 July 2025), which corresponds to the time window in which this study was conceived and originally conducted. May corresponds to the earliest notification of locally acquired cases in mainland France during the 2025 outbreak [25], while July marks the point at which this study was originally conducted with the aim of supplementing surveillance through real‑time inference [26]. Although locations with outbreak notifications beyond July were not included in this analysis, the proposed framework remains fully applicable to outbreaks notified later in the season (e.g., Bergerac [12]).

**Fig 1:**
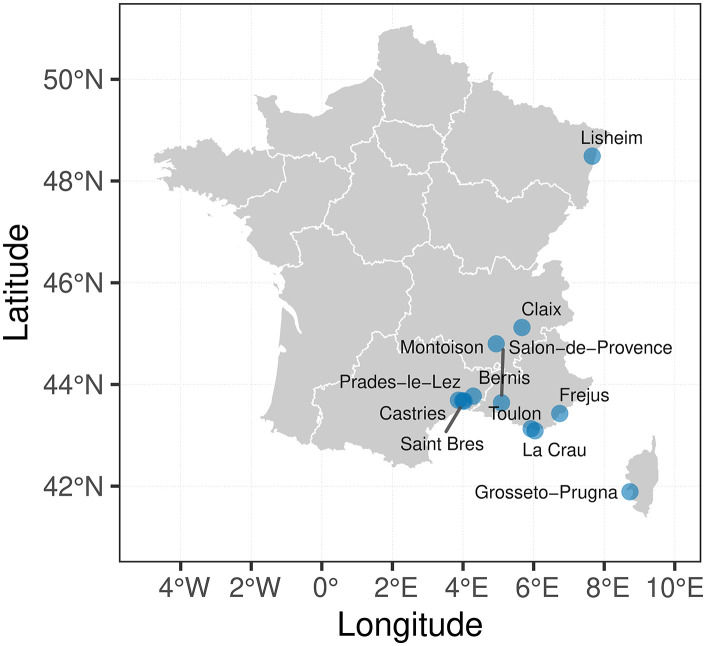
French municipalities with autochthonous chikungunya cases reported by the end of July 2025 [27]. Administrative boundaries are from the GADM database (https://gadm.org/data.html) and were accessed via the geodata::qadm() function in **R.**

Although 79 locations were ultimately affected during the 2025 chikungunya outbreak [3], only 12 of these had reported cases during the early phase ((1 May to 22 July 2025) [25], which corresponds to the period in which this study was conceived. Between 1 May and 22 July 2025, chikungunya outbreaks were notified in the following French municipalities (department, region), all of which were included in our analysis (Fig 1): La Crau (Var, Provence-Alpes-Côte d’Azur), Prades-le-Lez (Hérault, Occitanie), Salon-de-Provence (Bouches-du-Rhône, Provence-Alpes-Côte d’Azur), Grosseto-Prugna (Corse-du-Sud, Corse), Montoison (Drôme, Auvergne-Rhône-Alpes), Bernis (Gard, Occitanie), Lipsheim (Bas-Rhin, Grand Est), Claix (Isère, Auvergne-Rhône-Alpes), Fréjus (Var, Provence-Alpes-Côte d’Azur), Saint-Brès/Castries (Hérault, Occitanie), and Toulon (Var, Provence-Alpes-Côte d’Azur).

### Model initialisation and simulation setup

The application of the proposed framework requires three location-specific inputs: (i) climate time series (temperature, precipitation, and evaporation), (ii) human population density at 4 km^2^ resolution, and (iii) the date of introduction of an infectious individual into a fully susceptible population.

Daily temperature, precipitation, and evaporation data were obtained from the ERA5-Land climate reanalysis dataset, accessed via the Copernicus Climate Data Store [28], covering the period from January 1, 2015, to June 13, 2025. For the remainder of 2025, climate conditions were approximated using historic daily values from 2015-2024, generating ten climate scenarios to represent plausible future climatic conditions for 2025. Human population density was sourced from the gridded population dataset derived from the 2021 Population and Housing Census, available through the Eurostat web portal [29]. The climate data have a spatial resolution of 0.1° × 0.1°, and the population data have a resolution of 1 km²; both were resampled to the model’s operational grid of 4 km².

For each municipality, the operational grid of the model was defined by identifying the 4 km² grid cell(s) containing most of the population and assuming the presence of the index case within the corresponding cell. Except for larger municipalities, Salon‑de‑Provence (4 grid cells), Fréjus (2 grid cells), and Toulon (4 grid cells), the population in all other locations was concentrated within a single operational grid cell. For the larger municipalities, we simulated the model independently for each grid cell, using the population density within that cell, climatic variables were extracted at the cell’s centroid, and assuming the index case to be located within that cell. Subsequently, we averaged the outputs across these grid cells to characterise the key epidemiological features for Salon‑de‑Provence, Toulon, and Fréjus.

Given the limited epidemiological information and uncertainty during the early phase of the outbreaks, we used the reported date of the index case—the first case notified by public health authorities [30]—as the date of introduction of an infectious individual into a susceptible population. These introduction dates were obtained from the weekly bulletin of the French national public health agency [25,27], and were used to initialise the outbreaks in the model simulations.

For three of the outbreak locations (Salon-de-Provence, Castries, and Grosseto-Prugna), weekly epidemic data were available [25,27]. As these were the only locations with sufficiently detailed early‑phase data, we further evaluated the model against the cumulative number of cases reported by public health authorities for these locations. At these locations, outbreaks were initialised using both the reported date of the index case and a range of earlier candidate dates, representing plausible approximations of the unobserved primary case. Because climatic noise in the input time series introduces environmental stochasticity into the model, different outbreak‑initialisation dates generate distinct outbreak trajectories. To approximate the likely date of the primary case, we systematically shifted the index‑case date backward in time and selected the optimal date by minimising the root mean square error (RMSE) between predicted cumulative cases and observed data reported up to around 30 July 2025 [25]. This optimisation‑based approach provides a point estimate of the likely primary‑case date but does not explicitly quantify uncertainty.

For each French municipality included in the analysis (Fig 1), we quantified the real-time transmission dynamics by estimating (i) the outbreak onset or take-off window—defined as the interval beginning when the simulated daily number of new autochthonous cases first exceeds one and continues to increase until the outbreak reaches its peak, marking the initial amplification phase, (ii) average peak incidence, measured as the mean number of daily cases at the epidemic peak, and (iii) the final outbreak size, defined as the total cumulative number of cases by the end of the transmission season.

## Results

### Real-time transmission dynamics of chikungunya outbreaks

**Fig 2:**
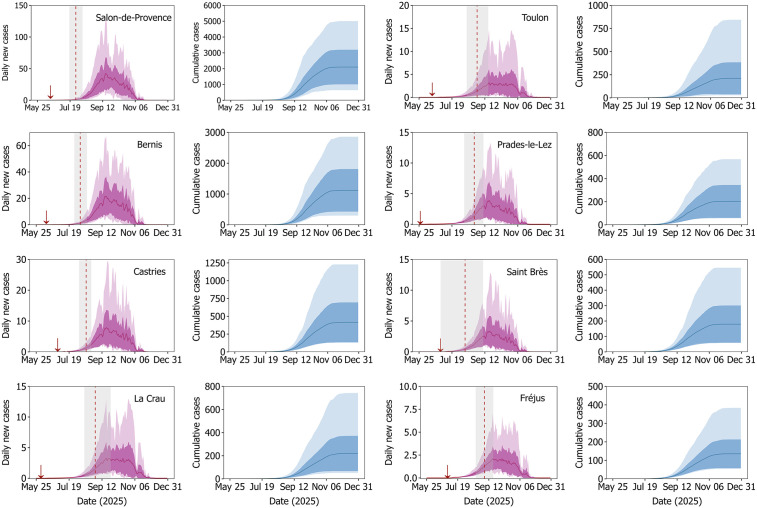
CHIKV transmission dynamics for municipalities in mainland France where large outbreaks are predicted. For each municipality in the figure, the red curves represent the simulated daily number of cases. The vertical red dashed lines indicate the estimated mean onset date of the outbreak, and the surrounding grey bands show the range between the earliest and latest estimated onset dates. The blue curves show the cumulative number of cases. In all plots, the solid lines represent mean values, dark ribbons indicate the 95% confidence intervals (CIs), and light-coloured bands correspond to the absolute maximum and minimum values. The introduction dates marked by red downward arrows correspond to the first reported symptomatic case in each municipality (Table 1). Variability in the predictions reflects stochasticity in climate variables—temperature, precipitation, and evaporation—for the remainder of the year 2025. See Fig B in S1 Text for municipalities where the CHIKV outbreak severity is predicted to be low.

**Fig 3:**
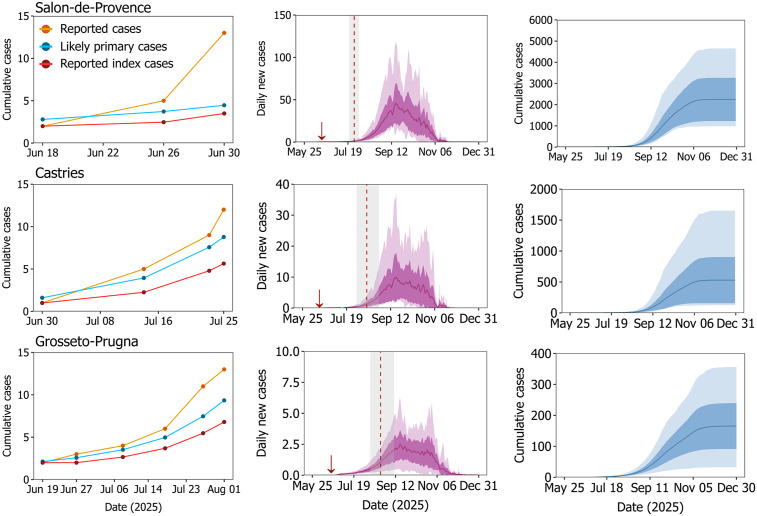
Early phase of chikungunya transmission in Salon-de-Provence (13 cases by 30 June 2025), Castries (12 cases by 25 July 2025), and Grosseto-Prugna (coastal) (13 cases by 1 August 2025). The leftmost panel shows reported data up to 1 August 2025 in orange [25]. The blue and red lines represent model outputs generated using the estimated dates of introduction of the infectious individual (i.e., the likely primary case) dates and the reported index-case dates (listed in Table 1). The inferred introduction dates were 29 May 2025 for Salon-de-Provence, 15 June 2025 for Castries, and 7 June 2025 for Grosseto-Prugna. In the middle column, the red plots show the daily number of cases; the vertical red dashed lines indicate the estimated mean outbreak onset date, and the surrounding grey bands denote the range between the earliest and latest estimated onset dates. The right column shows cumulative case count in blue. In all panels, the solid lines represent the mean values, the dark ribbons indicate the 95% confidence intervals (CIs), and the light-coloured bands correspond to the absolute maximum and minimum values. The estimated introduction dates are marked by red downward arrows.

We simulated the model with one symptomatic index case for all the municipalities (Fig 1), except for Salon-de-Provence and Grosseto-Prugna, where the model was initialised with two index cases based on the early-phase epidemiological information [31]. The early-phase simulations, in the absence of any control or mitigation measures, indicated that the larger outbreaks would occur in Salon-de-Provence (2,102 cases; 95% CI: 1,009.5–3,196.0), Bernis (1,119.6 cases; 95% CI: 430.0–1,809.2), Castries (413.8 cases; 95% CI: 132.6-695.1), La Crau (220.0 cases; 95% CI: 68.0–372.2), Toulon (211.0 cases; 95% CI: 76.0–384.2), Prades-le-Lez (203.0 cases; 95% CI: 60.6–344.9), Saint-Brès (180.4 cases; 95% CI: 52.4–301.3), Grosseto-Prugna (167.5 cases; 95% CI: 39.0–332.8), and Fréjus (135.6 cases; 95% CI: 33.3–213.2) (Figs 2 and 3). Smaller outbreaks were inferred for the remaining municipalities (Table 1).

**Table 1:** Outbreak locations in France with geographic and demographic information, together with model-inferred estimates for the outbreak onset window, average peak incidence (mean number of daily cases at peak), projected final outbreak size (total cumulative number of cases by the end of the epidemic), and reported final size [3]. Values in parentheses in the reported final outbreak size column denote the 95% confidence intervals (CIs), while the dates in parentheses in the outbreak onset window column indicate the range between the earliest and latest estimated onset dates. Symptom onset dates for the index case correspond to those reported through epidemiological surveillance as of July 2025 [25].

Region	Department	Municipality	Symptom onset date for the index case	Lat/Lon	Estimated population (per 4 km^2^)	Outbreak onset window	Average peak incidence (Date)	Projected final outbreak size	Reported final outbreak size [3]
Provence- Alpes-Côte d’Azur	Bouches-du-Rhône	Salon-de-Provence	16/06/2025	(43.64, 5.09)	8,525	28/07/2025 (17/07/2025-09/08/2025)	54.1 (20/09/2025)	2,102.7 (1,009.5-3,196.0)	12
Occitanie	Gard	Bernis	11/06/2025	(43.77, 4.29)	7,000	06/08/2025 (27/07/2025- 17/08/2025)	27.8 (24/09/2025)	1,119.6 (430.0-1,809.2)	1
Occitanie	Hérault	Castries	30/06/2025	(43.68, 3.99)	6,014	16/08/2025 (04/08/2025-25/08/2025)	9.9 (24/09/2025)	413.8 (132.6-695.1)	17
Provence- Alpes-Côte d’Azur	Var	La Crau	02/06/2025	(43.09, 6.04)	10,637	31/08/2025 (13/08/2025- 26/09/2025)	4.3 (02/10/2025)	220.0 (68.0-372.2)	2
Provence- Alpes-Côte d’Azur	Var	Toulon	16/06/2025	(43.13, 5.93)	31,997	30/08/2025 (12/08/2025-17/09/2025)	4.0 (28/09/2025)	211.0 (76.0-384.2)	1
Occitanie	Hérault	Prades-le-Lez	27/05/2025	(43.69, 3.87)	6,000	25/08/2025 (08/08/2025- 09/09/2025)	4.8 (20/09/2025)	203.0 (60.6-344.9)	1
Occitanie	Hérault	Saint-Brès	30/06/2025	(43.67, 4.03)	4,094	26/08/2025 (10/08/2025-09/09/2025)	4.2 (20/09/2025)	179.7 (58.4-301.1)	0^*^
Provence- Alpes-Côte d’Azur	Var	Fréjus	01/07/2025	(43.43, 6.74)	12,975	04/09/2025 (19/08/2025-19/09/2025)	2.7 (26/09/2025)	135.6 (33.3-213.2)	84
Corse	Corse du Sud	Grosseto-Prugna	19/06/2025	(41.89, 8.73)	3,173	28/08/2025 (09/08/2025- 10/09/2025)	3.1 (28/09/2025)	167.5 (39.0-332.8)	14
Auvergne-Rhône-Alpes	Drôme	Montoison	13/06/2025	(44.80, 4.94)	1,460	NA	0.1 (11/09/2025)	4.4 (1.3-7.4)	3
Grand Est	Bas-Rhin	Lipsheim	26/06/2025	(48.49, 7.66)	2,643	NA	0.04 (08/09/2025)	2.0 (1.2-3.0)	1
Auvergne-Rhône-Alpes	Isère	Claix	01/07/2025	(45.12, 5.67)	6,000	NA	0.01 (13/08/2025)	0.5 (0.2-0.8)	5

*The index case was initially suspected to be in Saint‑Brès/Castries [27]. Subsequent epidemiological confirmation indicated that the case occurred in Castries, and Saint‑Brès remained unaffected [25].

Across the municipalities, we inferred both the possibility of sustained outbreaks and the possibility of no outbreak-amplification. A subset of municipalities, i.e., Salon‑de‑Provence, Bernis, Castries, La Crau, Toulon, Prades‑le‑Lez, Saint‑Brès, Fréjus, and Grosseto‑Prugna, showed conditions conducive to sustained transmission, with model‑inferred outbreak sizes substantially larger than one hundred cases (Table 1). In contrast, other municipalities exhibited very low outbreak potential, with no indication of escalation of transmission chains and simulated daily incidence remaining below one case throughout the season (e.g., Montoison, Lipsheim, and Claix in Table 1).

Among the municipalities with the possibility of sustained transmission and substantially larger outbreak sizes, the inferred onset window showed marked temporal variation (Table 1). The initial amplification phase could begin as early as 17 July 2025 in Salon‑de‑Provence, while in Fréjus it was inferred to start as late as 19 August 2025. Across these municipalities, the onset window could extend well into late September, with La Crau showing the latest inferred take‑off, lasting until 26 September 2025. Consistent with these differences in onset timing, the peak incidence was inferred to occur earliest in Salon‑de‑Provence (20 September 2025) and latest in La Crau (2 October 2025) (Table 1). Similarly, the magnitude of peak incidence varied substantially across locations, ranging from approximately 54 daily cases in Salon‑de‑Provence to approximately 3 daily cases in Fréjus (Table 1), highlighting pronounced spatial heterogeneity across the municipalities.

Model simulations showed good agreement with the observed case counts available during the early phase of infections in Salon‑de‑Provence (13 cases by 1 August 2025), Castries (12 cases by 27 July 2025), and Grosseto‑Prugna (coastal) (Fig 3). For these municipalities, we also inferred the likely dates of primary infection, which consistently preceded the reported index‑case symptom onset dates. The inferred primary infection dates were 7 June 2025 for Grosseto‑Prugna (compared with the reported index case on 19 June 2025), 15 June 2025 for Castries (compared with 30 June 2025), and 29 May 2025 for Salon‑de‑Provence (compared with 16 June 2025). Overall, these inferred dates showed better agreement with the observed early‑phase epidemic trajectories than the reported index‑case dates alone (Fig 3).

## Discussion

An unprecedented total of 809 locally transmitted (autochthonous) chikungunya cases were reported in mainland France during the enhanced surveillance period (May–November 2025) [3], underscoring the reality of an emerging epidemic threat of chikungunya transmission in France and, more broadly, across parts of Europe. In France, public‑health policy for the prevention and control of chikungunya is grounded in routine entomological and epidemiological surveillance, which guides vector control activities, the mobilisation of public‑health professionals, and public awareness campaigns [4]. In practice, surveillance and control rely on a wide range of actions, including epidemiological investigations, door‑to‑door activities for active case finding, entomological surveys, elimination of larval breeding sites, night‑time adulticide treatments, and raising public awareness and increasing the use of personal protective measures. All these interventions could benefit from improved targeting in space and time to increase their effectiveness and maximise scarce public health resources.

Despite enhanced surveillance being in place, uncertainties around detection delays, underreporting and the timing of subsequent mitigation efforts remain unavoidable—particularly during the early phase of an outbreak. For example, although enhanced surveillance in France began on 1 May 2025, when chikungunya importations from La Reunion, Mauritius, Madagascar and other affected regions were already at their peak, there was still a gap of almost one month before the first autochthonous case was detected on 27 May 2025 [3]. The underlying interaction between mosquito population dynamics and viral transmission during the gap between importation and the first notified autochthonous case cannot be fully understood from surveillance data alone, yet this period plays a critical role in shaping early transmission dynamics and the effectiveness of interventions.

From the detection of this first autochthonous case to 15 July 2025, a total of 31 locally transmitted cases were reported in 13 municipalities across six regions (Fig 1). We captured this same early transmission window in the present study. Using the early epidemiological information available for these locations [27], we inferred three key epidemiological indicators in real-time: the relative outbreak risk across municipalities, the likely window for initial amplification and the potential magnitude of outbreaks.

We inferred substantial variability in these epidemiological indicators across locations. The onset of the initial amplification phase was inferred to vary from late July to early September 2025, and several locations showed no evidence of entering an initial amplification phase of transmission (Table 1). The latest epidemiological data available in the public domain do not categorise onset timing for the affected locations, preventing direct comparison. However, recent surveillance reports indicate that transmission ended by mid‑November [3], which is consistent with the inferred transmission trajectories across all considered locations (Figs 2 and 3), supporting the internal coherence of the modelled transmission dynamics.

Most locally transmitted outbreaks did not develop into prolonged transmission chains and therefore did not result in large outbreaks, suggesting that early detection and control measures were generally effective. In Fréjus, one of the worst-affected locations included in this study, a sustained local transmission persisted for more than three months, with 84 autochthonous cases reported [3]. Our model inferred an outbreak magnitude of 136 autochthonous cases and a transmission duration of approximately three to four months for this location (Fig 2; Table 1), consistent with the observed duration and intensity of transmission [3]. Given that the outbreak in Fréjus was detected early, when control efforts are typically most effective, further investigation into the factors underlying its unusually prolonged transmission may be warranted.

Several transmission events were detected early through the surveillance system, enabling timely implementation of control measures by the ARS and their mosquito control operators [3]. Many of these events—such as those in Salon‑de‑Provence, Bernis, Castries, La Crau, and Toulon (Table 1)—had the potential for larger outbreaks as inferred by our modelling study, and timely public-health and vector-control efforts contributed to preventing amplification at these locations. In contrast, in several other locations, including Montoison, Lipsheim and Claix (Table 1), our model inferred only a small number of cases and a low potential for outbreak amplification, which is consistent with more recent epidemiological reports [32]. The limited outbreak potential inferred for these locations likely reflects underlying environmental conditions that were not conducive to sustained transmission, rather than the effect of prevention and control interventions alone.

This difference is exemplified by locations where the index case was reported on the same date (01/07/2025), such as Fréjus and Claix (Table 1). Despite identical case information at the time of detection, the model predicted markedly different outbreak trajectories: approximately 135 cases in Fréjus and fewer than 1 case in Claix (Table 1).

The subsequent epidemiological investigations also highlight the uncertainty around the confirmation and detection of index cases. For example, Claix illustrates uncertainty related to the timing of the index case and the possibility of under-reporting. Using the reported index case on 1 July 2025, our model predicted fewer than one case (Table 1). However, simulation with later index cases, from around 8 July onwards, inferred the possibility of three to five cases (Fig E in S1 Text). This matches the number of reported cases (Table 1) and suggests that under-reporting or undetected infections may have contributed.

Predicted heterogeneity in outbreak potential across locations highlights the value of mechanistic inference in supplementing routine surveillance by identifying where transmission is unlikely to amplify and where a proportionally more intensive response may be warranted. This supports targeted intervention and vector‑control programmes conducted by ARS and their mosquito control operators. Since the vector‑control activities are typically initiated following case detection, mechanistically inferred outbreak risk can help guide the intensity of these control efforts in proportion to the predicted risk and serves as a prioritisation tool for intervention areas, particularly in context where field resources are limited.

Even with a well‑functioning surveillance system and early detection of infections, the possibility of undetected cases preceding the reported index case—and delays in the medical confirmation of that case—can have direct implications for the effectiveness of preventive and control efforts. The combined delay between detection, confirmation, and the initiation of vector‑control activities can shift the perceived timing of the outbreak, reducing the impact of interventions (e.g., Bergerac [12]). Such temporal misalignment, driven by both reporting delays and unobserved early infections, can result in control measures being deployed later in the epidemic trajectory than assumed. These dynamics underscore the value of real‑time mechanistic inference for synchronising control efforts with the actual, rather than the observed, progression of transmission.

The proposed real‑time modelling framework has been successfully evaluated across multiple historic outbreaks, consistently identifying the start of transmission chains, the onset window of the initial amplification phase of transmission, the timing of the transmission peak, the end of transmission, and the overall duration of outbreak events without requiring back‑fitting (e.g., [9,12,22]). However, the framework remains sensitive to environmental stochasticity, short‑term climatic fluctuations, and uncertainties in introduction timing and the spatial location of the index case, all of which can influence the inferred magnitude of an outbreak, even though the inferred timings remain robust. When informed with real‑time surveillance data, the framework can also generate more reliable estimates of outbreak magnitude, as demonstrated in previous applications (i.e., Bergerac [12]; Fano and Lodi [22]). At the same time, the current implementation does not explicitly incorporate control interventions or the potential contribution of passive dispersal (e.g., via transport networks, human mobility, or trade routes), and therefore, cannot track their effects in real-time. These represent mechanistic components that could be readily and more explicitly integrated into the framework in future developments.

As chikungunya and other *Aedes*‑borne viruses continue to expand across Europe [33,34], approaches that combine routine surveillance with real‑time mechanistic modelling will be increasingly important for strengthening preparedness, guiding rapid response, and improving the efficiency of prevention and control programmes. Unlike post‑outbreak analyses (e.g., [13,14,17,19,20]), which are typically conducted months or years after an event and provide valuable epidemiological insight but cannot inform ongoing surveillance, real‑time inference offers operationally relevant information during the period when interventions can still alter the course of transmission. As the development cycles of both the vector and the pathogen are tightly constrained by climatic conditions, our climate‑sensitive and well‑evaluated mechanistic framework can robustly and rapidly quantify (within 24–48 hours of a notification) the progression of transmission for potential outbreak locations and the population clusters associated with them. Such quantification directly informs how transmission chains unfold over time and can support surveillance systems in designing evidence‑based, targeted control strategies aimed at preventing or limiting the establishment of sustained transmission cycles.

## Conclusion

Our results show that real‑time inferences generated by a climate‑sensitive mechanistic framework tailored to the chikungunya–*Ae. albopictus* system provide robust estimates of key epidemiological indicators, including the start of transmission chains, the likely window of the amplification phase, the timing of the transmission peak, the end of transmission, the overall duration of outbreak events, and the potential magnitude of outbreaks ahead of the transmission season. When combined with real‑time data from routine surveillance, this approach offers early, evidence‑based insight into heterogeneity across outbreak locations, vector population dynamics, and the development delays inherent to the vector–pathogen system. Such real‑time situational awareness can support decision‑makers in designing targeted, timely vector-control and prevention strategies—both before and during an ongoing outbreak—thereby strengthening the capacity to limit or prevent the establishment of sustained transmission cycles. The framework can be implemented operationally by providing alert thresholds for early action, updating the temporal progression of an outbreak as new data arrive, and quantifying residual uncertainty relevant to decision‑making. These features can guide the intensity and prioritisation of vector‑control activities, particularly where field resources are limited. The study also highlights key research gaps, including improved characterisation of early under‑reporting and systematic evaluation of real‑time performance. Addressing these gaps will further strengthen the operational value of mechanistic inference for preventing the establishment of sustained transmission cycles.

## Supporting information

S1 Text Supplementary text containing full details of the model and its parameterisation, as well as additional results. (DOCX)
