# Supplementary material for: A real-time mechanistic framework for early inference of chikungunya transmission and outbreak sizes in mainland France, 2025
Source: PLoS Negl Trop Dis. 2026 Jul 13;20(7):e0014534. doi: 10.1371/journal.pntd.0014534 (PMC13375128; doi:10.1371/journal.pntd.0014534)
Supplement: S1 Text — (DOCX) [file pntd.0014534.s001.docx]

**S1 Text: Supplementary information**

**A real-time mechanistic framework for early inference of chikungunya transmission and outbreak sizes in mainland France, 2025**

**Authors:**

Sandeep Tegar^1,2^ (<https://orcid.org/0009-0003-2445-9860>), Dominic P. Brass^1^ (<https://orcid.org/0000-0002-4900-9124>), Bethan V. Purse^1^ (<https://orcid.org/0000-0001-5140-2710>), Antoine Mignotte^3^ (<https://orcid.org/0000-0001-7433-1994>), Guillaume Lacour^3^ (<https://orcid.org/0000-0002-9588-6064>), Christina A. Cobbold^2^ ([https://orcid.org/0000-0001-8814-7688)](https://orcid.org/0000-0001-8814-7688)2) & Steven M. White^1^ (<https://orcid.org/0000-0002-3192-9969>)

**Affiliations**

1. UK Centre for Ecology & Hydrology, Benson Lane, Wallingford, Oxfordshire, UK.
2. School of Mathematics and Statistics, College of Science and Engineering, University of Glasgow, Glasgow, UK.
3. Altopictus, Pérols, France.

**Corresponding author:** Sandeep Tegar ([santeg@ceh.ac.uk](mailto:santeg@ceh.ac.uk))

**Contents**

A. Modelling framework

B. Chikungunya transmission dynamics in small outbreak locations

C. Climate, population dynamics of *Aedes albopictus*, and chikungunya introduction dates

**References**

**A. Modelling framework**

The model used to study the population dynamics of *Aedes albopictus* and the transmission dynamics of the chikungunya virus (CHIKV) by *Ae. albopictus* is based on a modelling framework previously developed for studying the transmission dynamics of the dengue virus by *Ae. albopictus* [9]. Although originally developed for dengue, the framework can be extended to chikungunya transmission by *Ae. albopictus* provided that climate-sensitive epidemiological traits for the relevant vector-pathogen system are available. To achieve this, we modelled the thermal performance curves for the extrinsic incubation period (EIP; the time between a mosquito feeding on an infected human and becoming infectious) and vector competence of the CHIKV–*Ae. albopictus* system [10], re‑parameterised the original *Ae. albopictus*–dengue framework (Brass et al. [9]) for chikungunya transmission and verified that the resulting framework reproduced epidemiological features of historical chikungunya outbreaks in Europe (e.g., Tegar et al. [12]).

The model is described by a system of continuous-time delay differential equations and incorporates the trait and population dynamics of *Ae. albopictus*, including an additional phenotypic structure in the adult mosquito population that accounts for size variations (e.g., wing length). In this framework, a generic stage-structured population in class-$(i,j)$, $N_{i,j}\left( t \right)$, represents the density of individuals in life-stage $i$ and phenotypic class $j$ at time $t$, the rate of change of the population is described by the system of delay-differential equations:

$$\frac{dN_{i,j}(t)}{dt}=R_{i,j}\left( t \right)-M_{i,j}\left( t \right)-D_{i,j}\left( t \right),$$

for $i=1,\ldots, n$ and $j=1, \ldots, m$. Here, $R_{i,j}(t)$ and $D_{i,j}(t)$ represent instantaneous recruitment and death rates for class-$(i,j)$, respectively, while $M_{i,j}(t)$ represents the instantaneous rate of maturation from the class-$(i,j)$ to the life-stage $i+1$. The recruitment rate into life-stage $i=1$ is given by the following expression:

$$R_{1,j}\left( t \right)=\sum_{k=1}^{m} \left( w_{k,j}(\alpha(t))\sum_{v=1}^{n} \beta_{v,k}\left( t \right)N_{v,k}(t) \right),$$

for $j=1, \ldots, m$, where $w_{k,j}(\alpha(t))$ denotes the proportion of individuals transitioning from phenotypic class $k$ to phenotypic class $j$ at time $t,$ depending on food availability $\alpha\left( t \right),$ and $\beta_{v,k}(t)$ is the birth rate of individuals in class-$(v,k)$ [24]. Maturation out of class-$(i,j)$ corresponds to the fraction of individuals that were recruited in class-$(i,j)$ $\tau_{i,j}(t)$ time units ago and survived for the stage duration $\tau_{i,j}(t)$. Formally, this is given by the relation $M_{i,j}\left( t \right)=R_{i,j}\left( t-\tau_{i,j}\left( t \right) \right)S_{i,j}(t)$, where

$$S_{i,j}\left( t \right)=\exp\left( -\int_{t-\tau_{i,j}(t)}^{t} \delta_{i,j}(s)ds \right)$$

is the probability that an individual in class-$(i,j)$ survives to progress to life-stage $i+1$, $\delta_{i,j}(t)$ is death rate for individuals in class-$(i,j)$, and $D_{i,j}\left( t \right)=\delta_{i,j}\left( t \right)N_{i,j}(t)$. In this modelling framework, the duration of life-stage $i$, i.e., $\tau_{i,j}(t)$, also varies with time and is governed by:

$$\frac{d\tau_{i,j}(t)}{dt}=1-\frac{g_{i,j}\left( t \right)}{g_{i,j}\left( t-\tau_{i,j}\left( t \right) \right)},$$

where, $g_{i,j}(t)$ denotes the development rate through the class-$(i,j)$. In this modelling framework, the functions $\delta_{i,j}(t)$, $\beta_{i,j}\left( t \right)$, and $g_{i,j}(t)$ are defined by the biologically derived environment-trait relationships (detailed in [9]).

The mosquito model predicts the temporal population dynamics of the different life stages of *Ae. albopictus* and tracks the dynamics of adult phenotypes, characterised by their wing lengths, arising from a single aquatic habitat of fixed dimensions (Fig A). Location‑specific conditions are incorporated through input time series of local climatic variables (temperature, precipitation, evaporation and photoperiod). Adult mosquitoes oviposit eggs either onto the water surface or around the edges of the habitat, with the proportion laid around the edges increasing as water levels decline. Eggs deposited in or around the aquatic habitat are classified as either diapausing ($E_{D}$) or non‑diapausing ($E_{\gamma}$) phenotypes, determined by a maternal effect in response to cooling environmental temperatures and decreasing photoperiod. The production of cold resistant diapausing eggs is triggered when falling temperatures and photoperiod reach a critical threshold. Once development is complete, both diapausing and non‑diapausing eggs may enter a quiescent state ($E_{Q}$) to survive dry periods or may hatch immediately into larvae ($L$). Quiescent eggs remain dormant until inundated by precipitation, after which they hatch immediately. Egg development and survival are assumed to be temperature‑dependent, with diapausing eggs additionally influenced by photoperiod. The aquatic habitat varies only in response to temperature, accumulated precipitation and evaporation of standing water, and is otherwise identical across all locations.


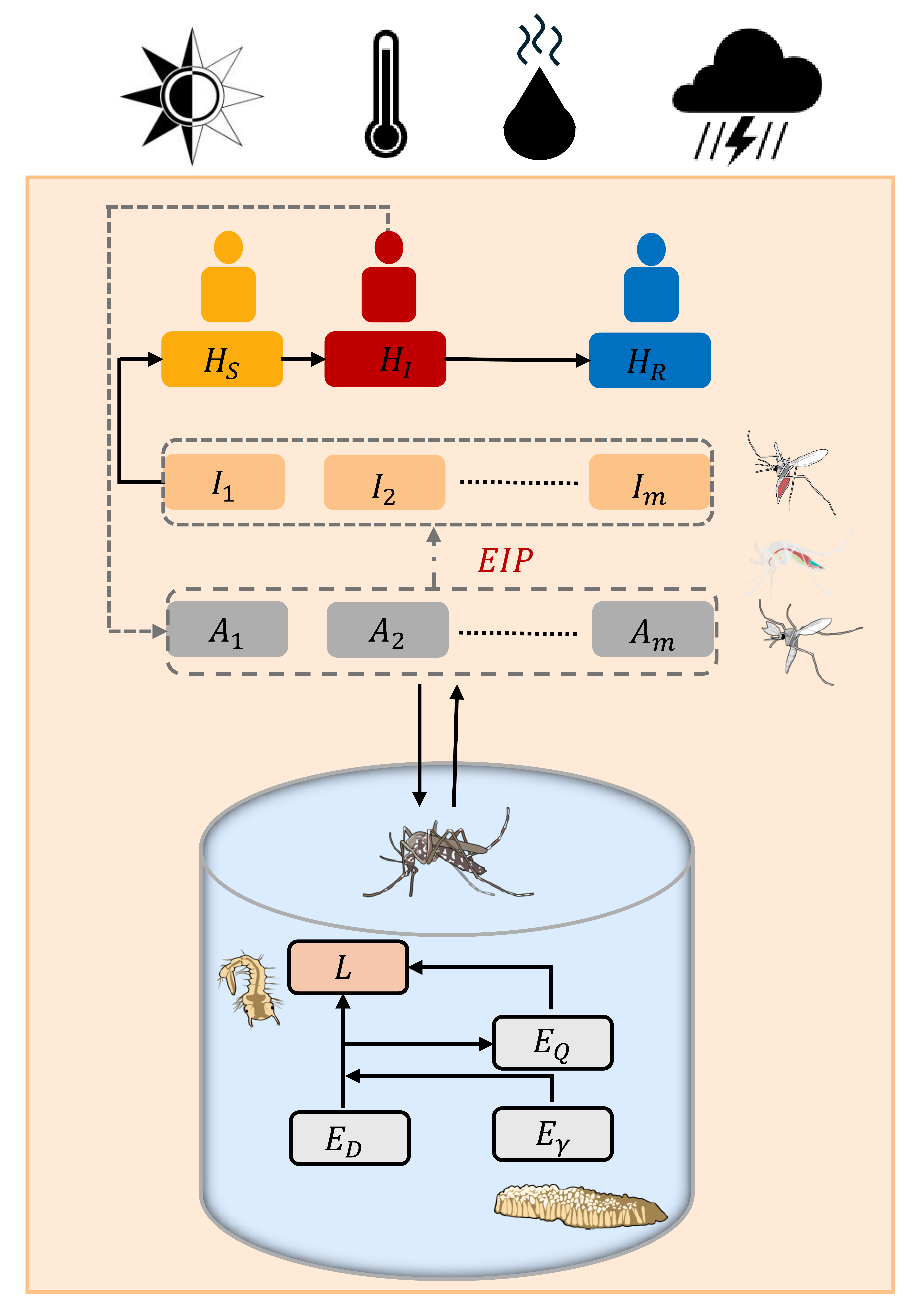


**Fig A.** A schematic of the climate-sensitive, stage and phenotypically structured epidemiological model. This model integrates the transmission dynamics of arboviruses by *Ae. albopictus* with the life-stage dynamics of the mosquito eggs (diapausing: $E_{D}$, non-diapausing: $E_{\gamma}$, and quiescent: $E_{Q}$), larvae ($L$), and adults (susceptible $A,$infectious $I$). The adult population is further partitioned according to adult wing length, into $m$ coexisting sub-classes denoted by subscript $i$, thereby establishing phenotypic structure in the adults. The infectious mosquito sub-class comprises adult mosquitoes from class $A_{i}$ that have taken an infected blood meal and then gone on to complete the extrinsic incubation period (EIP), defined as the average time required for the virus to incubate within the mosquito. The human population is represented by three compartments: susceptible ($H_{S}$), infected ($H_{I}$), and recovered ($H_{R}$). Graphic created by the authors using Microsoft PowerPoint as a drawing tool. No icons were obtained from Microsoft PowerPoint. Human and vaporisation icons were drawn by the authors. Weather and sun icons were obtained from Wikimedia Commons (<https://commons.wikimedia.org>), including Weather icons (<https://commons.wikimedia.org/wiki/File:Weather_Icons.png>; CC0) and Sun icon (<https://commons.wikimedia.org/wiki/File:Golden_Sun_Icon.svg>; CC0). Mosquito, egg raft, and larval illustrations were adapted from the NIAID NIH BioArt Source (<https://bioart.niaid.nih.gov>; CC0), which is in the public domain.

Larval mosquitoes ($L$) experience density‑dependent regulation through resource competition within the aquatic habitat, which is represented as a single larval class. The available resource is assumed to be consumed over the course of each day and replenished daily, maintained by temperature‑dependent metabolic processes in the larval environment. Upon completion of larval development, individuals pupate, with the pupal stage ($P$) modelled implicitly because it is not subject to density dependence. Aquatic habitats of fixed size (e.g., containers) are susceptible to flushing during heavy rainfall, whereby overflow causes larvae and pupae to be washed out. Flushing is represented by increased mortality of larvae and pupae whenever the water level exceeds the height of the container. These habitats may also dry out completely when all standing water evaporates, in which case all non‑quiescent juveniles are assumed to die.

Due to intraspecific competition for resources and the temperature dependence of development during the larval stage, adult mosquitoes ($A$) exhibit developmental plasticity. The average temperature and the average food available per larva per day over the course of the larval development period are used to predict the wing length at emergence. Adult phenotypic structure is represented by a large number of coexisting adult sub‑classes, each defined by its wing length ($A_{i}$, $i=1,\ldots, m$). Wing length determines both fecundity and longevity, with larger adults generally exhibiting higher reproductive output and longer lifespan. These traits are further assumed to vary with current temperature, meaning that adult performance reflects both historical environmental conditions experienced during larval development and the prevailing thermal environment at the adult stage.

The modelled population and trait dynamics of *Ae. albopictus* are extended to an epidemiological framework with susceptible–exposed–infected–recovered (SEIR) compartments for chikungunya virus transmission vectored by *Ae. albopictus* (Fig A). The adult mosquitos are partitioned in susceptible ($A_{i}$, $i=1,\ldots, m$) and infected ($I_{i}$, $i=1,\ldots, m$) individuals. The human population is partitioned into classes consisting of susceptible ($H_{S}$), infected ($H_{I}$) and recovered individuals who also become resistant to reinfection ($H_{R}$), with the exposed class modelled implicitly. The total human population density is assumed constant and is estimated from census data, as described in the main text.

Mosquitoes are assumed to bite at a temperature-dependent rate that is inversely proportional to the length of the gonotrophic cycle. The proportion of uninfected mosquitoes of a given wing length ($A_{i}$) that become infected ($I_{i}$) after biting an infected human ($H_{I}$) is temperature dependent. After a temperature-dependent extrinsic incubation period, an infected mosquito can bite and transmit the dengue virus to an uninfected human ($H_{S}$). After the intrinsic incubation period, the infected human can transmit the infection to new mosquitoes and recovers from the infection after a fixed recovery period.

The specific form of delayed differential equations, along with the mosquito environment-dependent parameters are fully described by Brass and colleagues [9].

To employ this eco-epidemiological framework to investigate the transmission of CHIKV by *Ae. albopictus*. we modelled the CHIKV specific temperature–trait relationships (i.e., thermal reaction norms) for key model parameters: the extrinsic incubation period (EIP)—the time required for the virus to replicate and reach the mosquito's saliva following ingestion of an infected blood meal—and the probability of CHIKV transmission from *Ae. albopictus* to humans (Table A) [10].

Additionally, the temperature–trait relationship for the probability of CHIKV transmission from humans to *Ae. albopictus* was adopted from Mordecai et al. [1]. Furthermore, the intrinsic incubation period (IIP)—the duration of CHIKV incubation within humans—was assumed to have an average value of 3 days [2], while the average recovery period from CHIKV infection was taken to be 7 days [3]. We have also evaluated the CHIKV model against historical chikungunya outbreaks in Italy and the 2025 outbreak France (e.g., Bergerac [12]). The results of the validation against the historic Italian outbreaks are part of a separate study and will be reported elsewhere.

**Table A.** Parameters for *Ae. albopictus*-chikungunya virus (CHIKV) transmission used in the modelling framework. Parameters specific to CHIKV transmission are listed here and the life-history traits of *Ae. albopictus* were adopted from the original modelling framework (Brass et al. [9]). In the table, $T$ denotes temperature.

| **Parameter** | **Values** | **Source** |
| --- | --- | --- |
| Temperature-dependent extrinsic incubation period (EIP) for CHIKV in *Ae. albopictus* | $55.03 exp({-0.13T}/{(1+\left( T/{273} \right))})$ | [10] |
| Temperature-dependent vector-to-human transmission probability | $0.0037\left( T-8.93 \right)\left( 34.73-T \right)$ | [10] |
| Temperature-dependent human-to-vector transmission probability | $0.0004394 T\left( T-3.62 \right)\left( 36.82-T \right)^{0.5}$ | [1] |
| Average intrinsic incubation period (IIP) of CHIKV in humans | 3 | [2] |
| Average recovery period in humans infected with CHIKV | 7 | [3] |

Model code is available on GitHub (https://doi.org/10.5281/zenodo.16783030).

**B. Chikungunya transmission dynamics in small outbreak locations**

This section presents the predicted CHIKV transmission dynamics for locations in France for which the model simulations predicted small chikungunya outbreaks: Lipsheim (Bas-Rhin, Grand Est), Saint-Chamond (Loire, Auvergne-Rhône-Alpes), Claix (Isère, Auvergne-Rhône-Alpes), Montoison (Drôme, Auvergne-Rhône-Alpes), and Grosseto-Prugna (Corse du Sud, Corse ; for the in-land location, this refers to a small settlement separated from the main coastal settlement).

| Lipsheim | 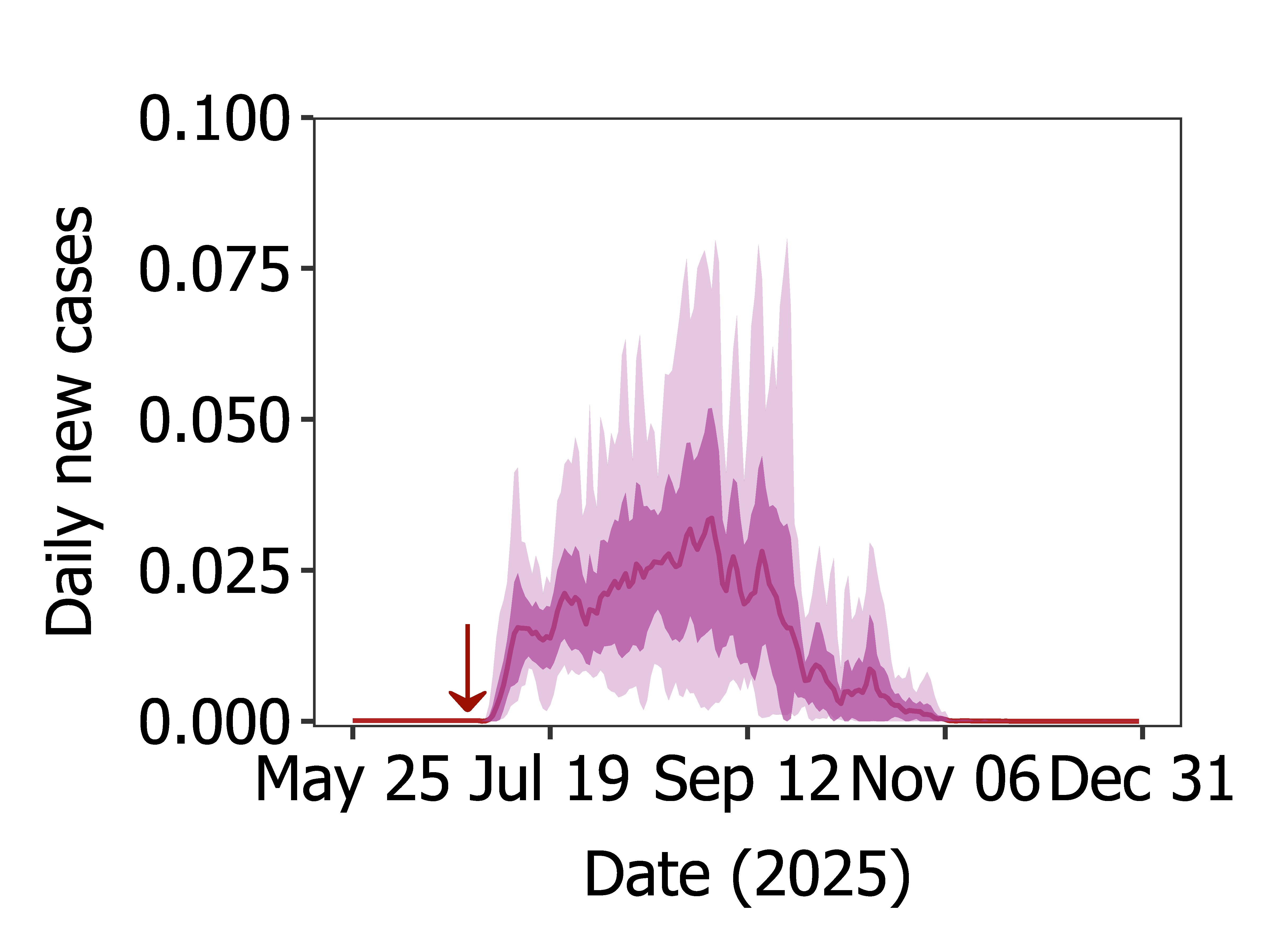 | 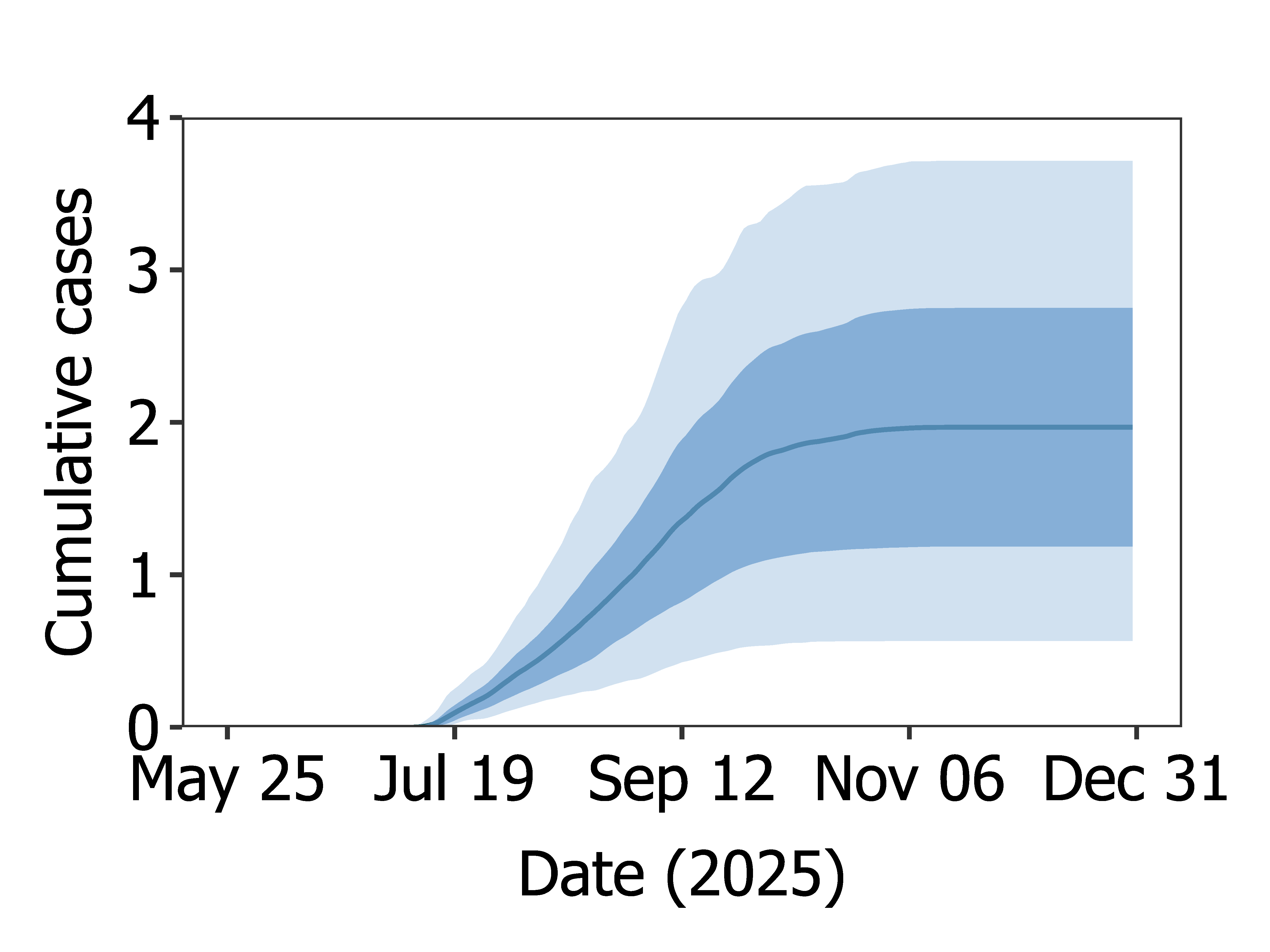 |
| --- | --- | --- |
| Saint-Chamond | 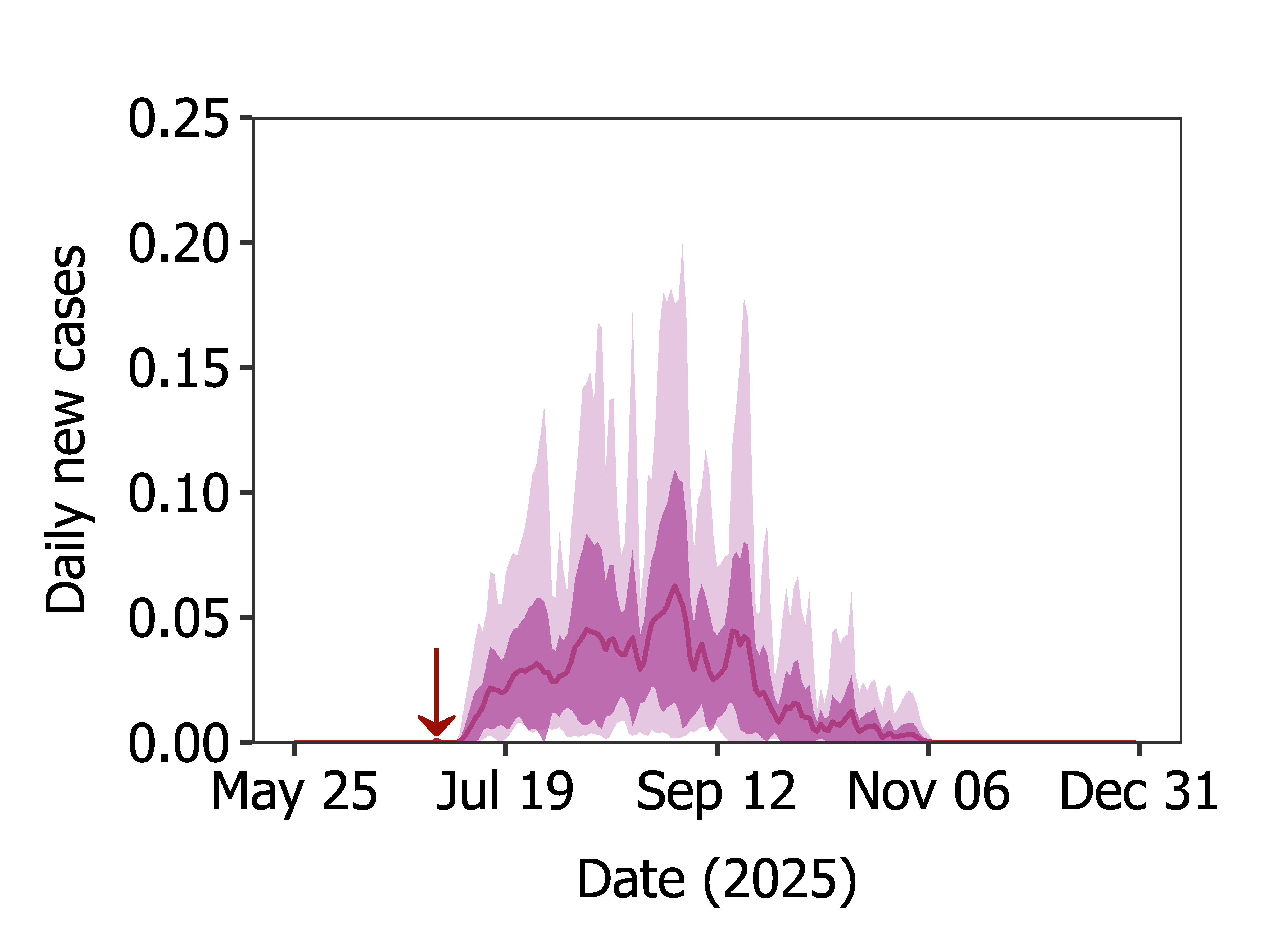 | 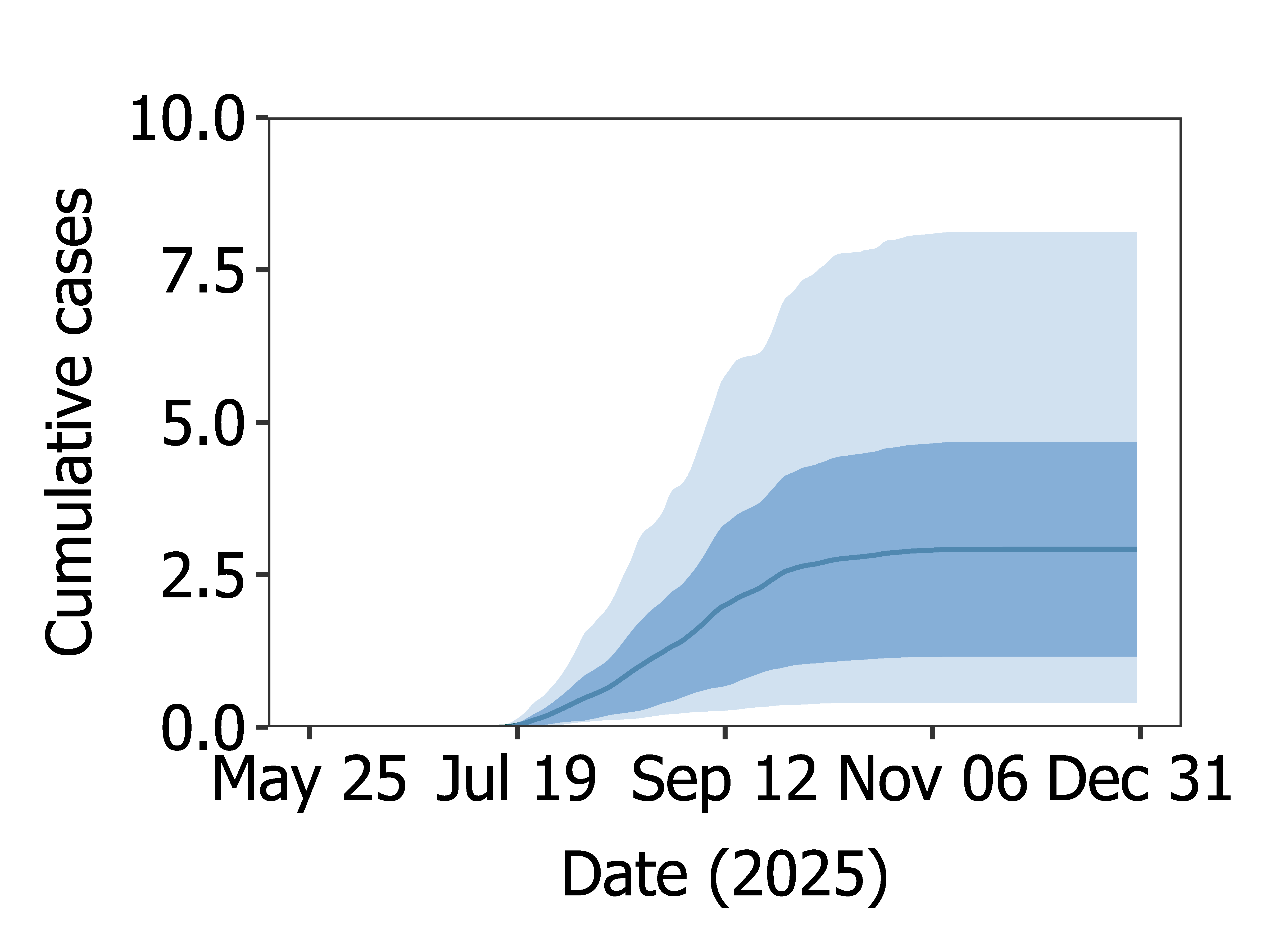 |
| Claix | 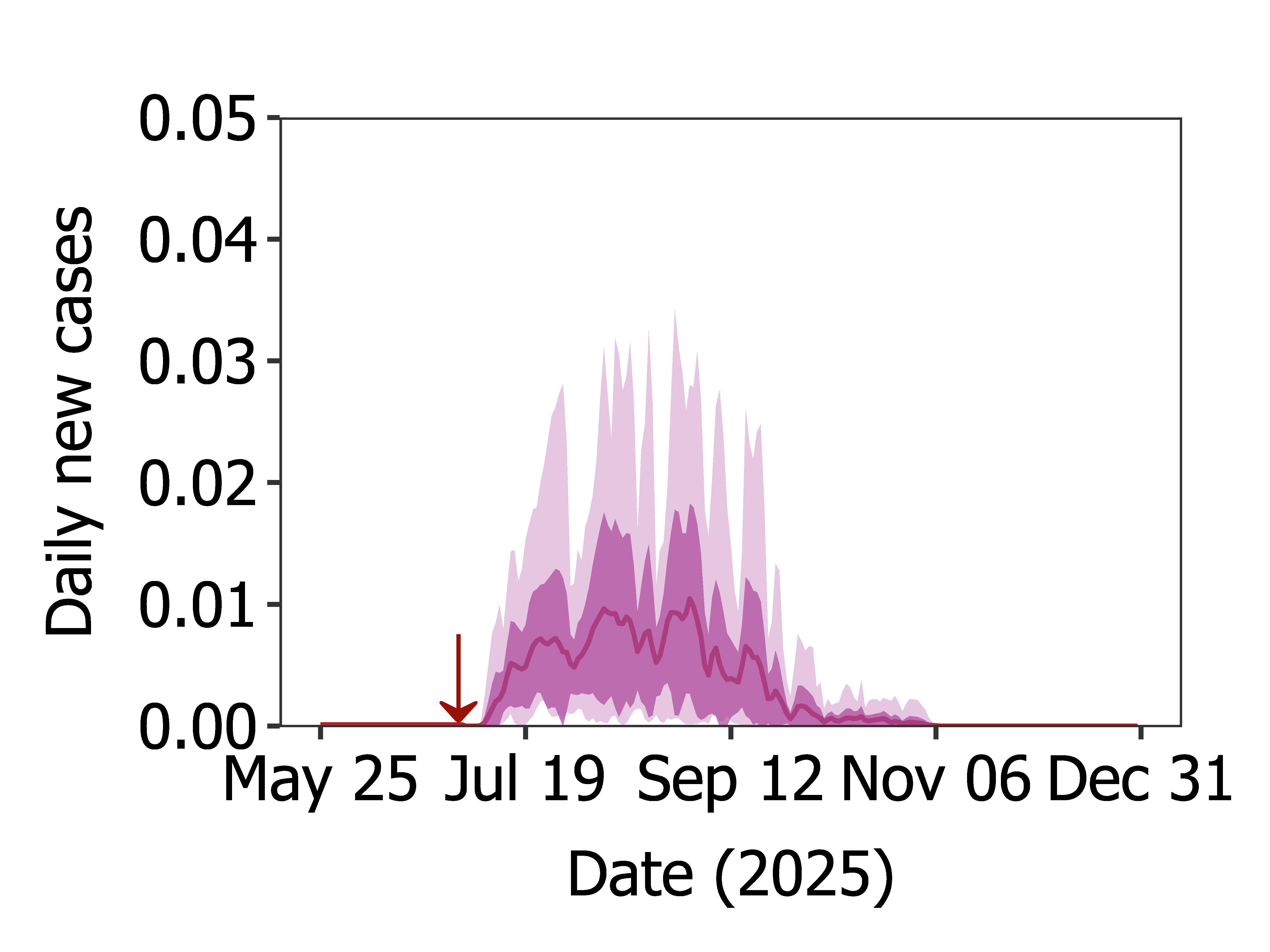 | 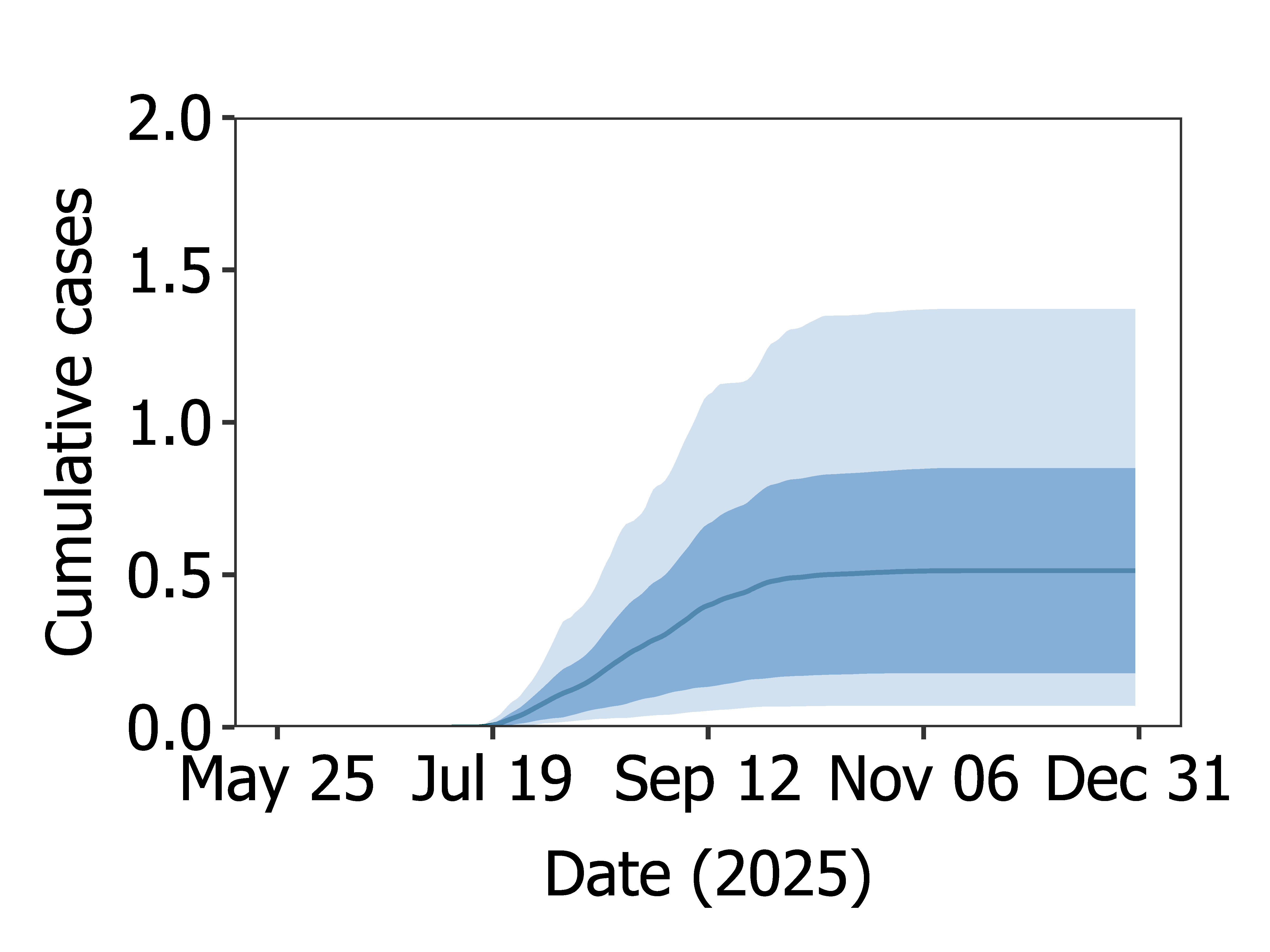 |

**Fig B (part 1).** CHIKV transmission dynamics for locations in France where smaller outbreaks are predicted. For each location shown in the panels above, the red plots represent the daily number of cases. The blue plots show the cumulative number of cases. In all plots, the solid lines represent the mean values, the dark ribbons indicate the 95% confidence intervals (CIs), and the light-coloured bands correspond to the absolute maximum and minimum values. The introduction dates, indicated by red downward arrows in the plots, correspond to the first reported symptomatic case in each location (Table 1). The variability in the projections reflects the estimated stochasticity in climate variables—temperature, precipitation, and evaporation—for the remainder of the year 2025, see section C for details.

| Montoison | 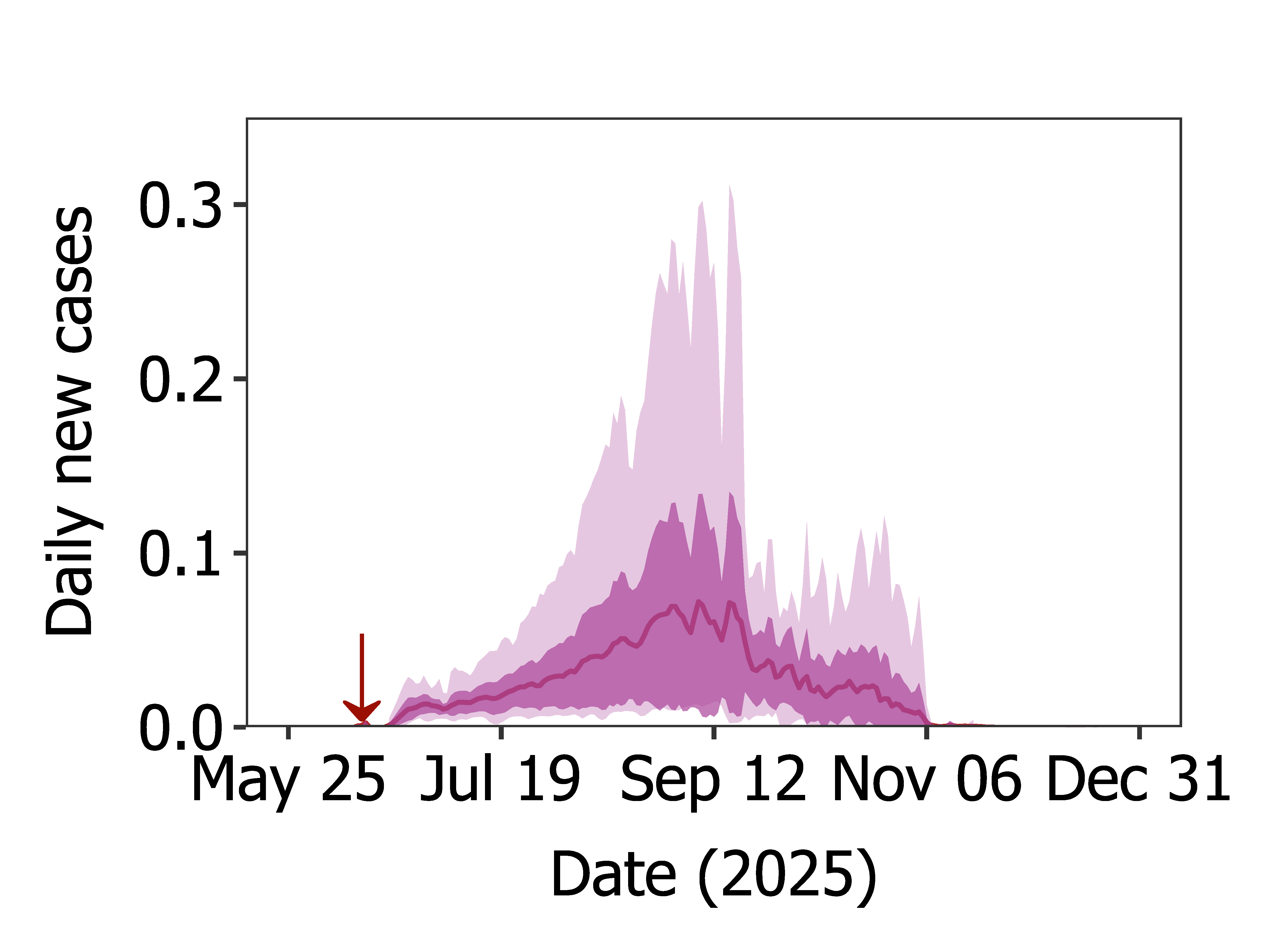 | 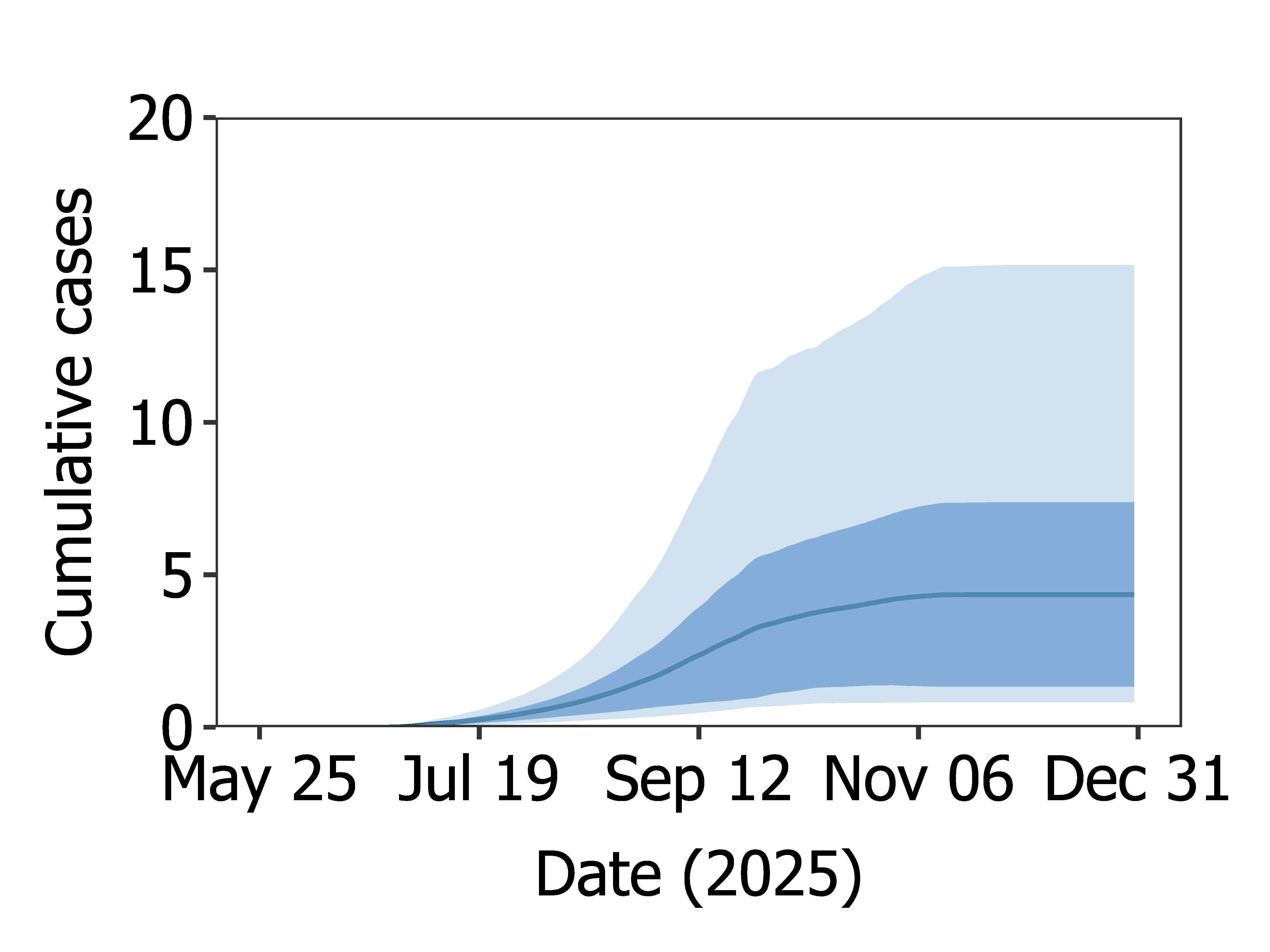 |
| --- | --- | --- |
| Grosseto-Prugna  (Inland) |  |  |
|  |  |  |

**Fig B (part 2).** CHIKV transmission dynamics for locations in France where smaller outbreaks could occur. For each location shown in the panels above, the red plots represent the daily number of cases. The blue plots show the cumulative number of cases. In all plots, the solid lines represent the mean values, the dark ribbons indicate the 95% confidence intervals (CIs), and the light-coloured bands correspond to the absolute maximum and minimum values. The introduction dates, indicated by red downward arrows in the plots, correspond to the first reported symptomatic index case in each location (Table 1). The variability in the projections reflects the estimated stochasticity in climate variables—temperature, precipitation, and evaporation—for the remainder of the year 2025, see section C for details.

**C. Climate, population dynamics of *Aedes albopictus*, and chikungunya introduction dates**

This section presents the climate scenarios (temperature and rainfall) considered for projecting the model forward in time beyond 13 June 2025, when, at the time of this study, recorded climate data ended. The climate scenarios are based on data from June 13 to December 31 from the previous ten years (2015–2024). Also presented are model estimated adult *Ae. albopictus* population dynamics, oviposition activities, and the total cumulative number of chikungunya cases at the end of epidemic (i.e., the final epidemic size) for each outbreak location reported up to 16 July 2025 (Table 1): La Crau (Var, Provence-Alpes-Côte d’Azur), Prades-le-Lez (Hérault, Occitanie), Salon-de-Provence (Bouches-du-Rhône, Provence-Alpes-Côte d’Azur), Grosseto-Prugna (Corse-du-Sud, Corse), Montoison (Drôme, Auvergne-Rhône-Alpes), Bernis (Gard, Occitanie), Lipsheim (Bas-Rhin, Grand Est), Claix (Isère, Auvergne-Rhône-Alpes), Fréjus (Var, Provence-Alpes-Côte d’Azur), Saint-Brès/Castries (Hérault, Occitanie), Toulon (Var, Provence-Alpes-Côte d’Azur, and Saint-Chamond (Loire, Auvergne-Rhône-Alpes).

Each of the panel plots in Fig C-O includes: (A) daily temperature, (B) daily total precipitation, (C) dynamics of adult and egg stages of *Ae. albopictus*, and (D) a boxplot of the final outbreak sizes (total cumulative chikungunya cases at the end of the season) for each introduction date, showing variation across ten climate scenarios from 2015 to 2024. Dots represent outliers in some studied cities, indicating exceptionally higher final sizes for specific climate scenarios, mostly corresponding to years warmer than the average annual temperature (e.g., 2022 for La Crau; Fig I). In subplots (A–C), the solid lines represent mean values, the dark ribbons indicate 95% confidence intervals (CIs), and the light-coloured bands correspond to the absolute maximum and minimum values.

**Bernis**


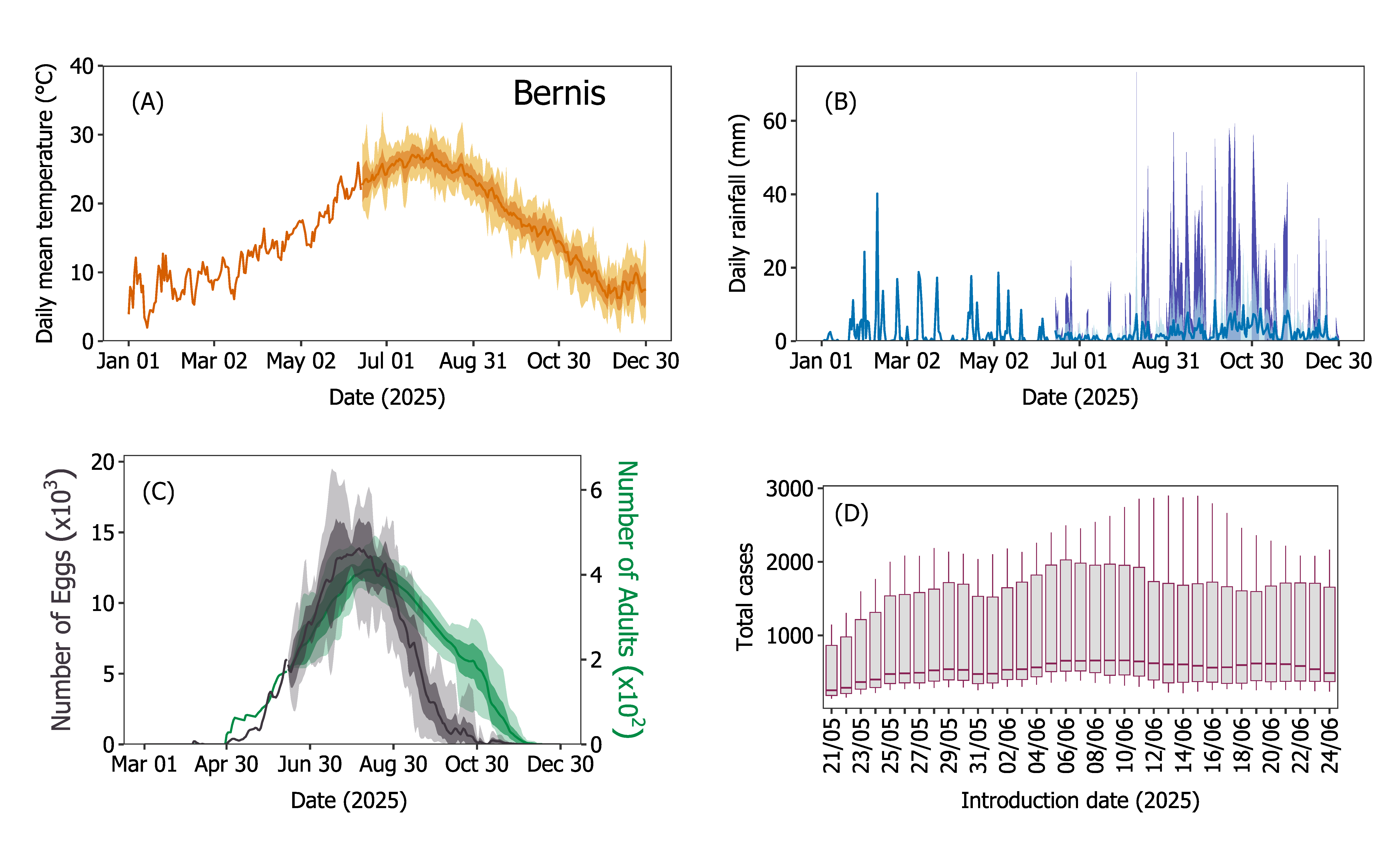


**Fig C.** Temperature, rainfall, adult *Ae. albopictus* dynamics with oviposition activity, and total predicted cases estimated for introduction dates spanning 3 weeks before to 2 weeks after the first reported symptomatic index case in Bernis.

**Castries**


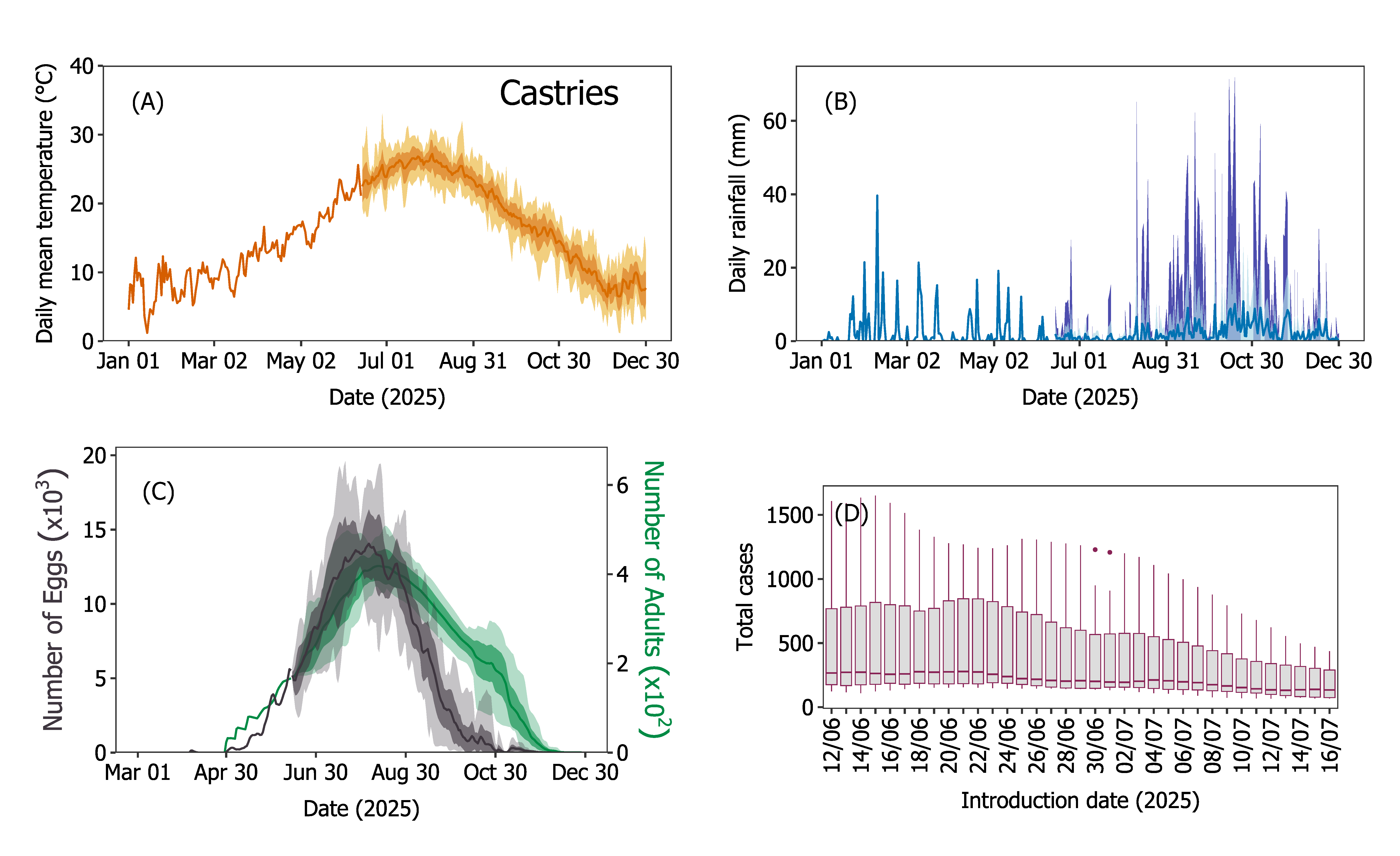


**Fig D.** Temperature, rainfall, adult *Ae. albopictus* dynamics with oviposition activity, and total predicted cases estimated for introduction dates spanning 3 weeks before to 2 weeks after the first reported symptomatic index case in Castries. Dots represent outliers.

**Claix**


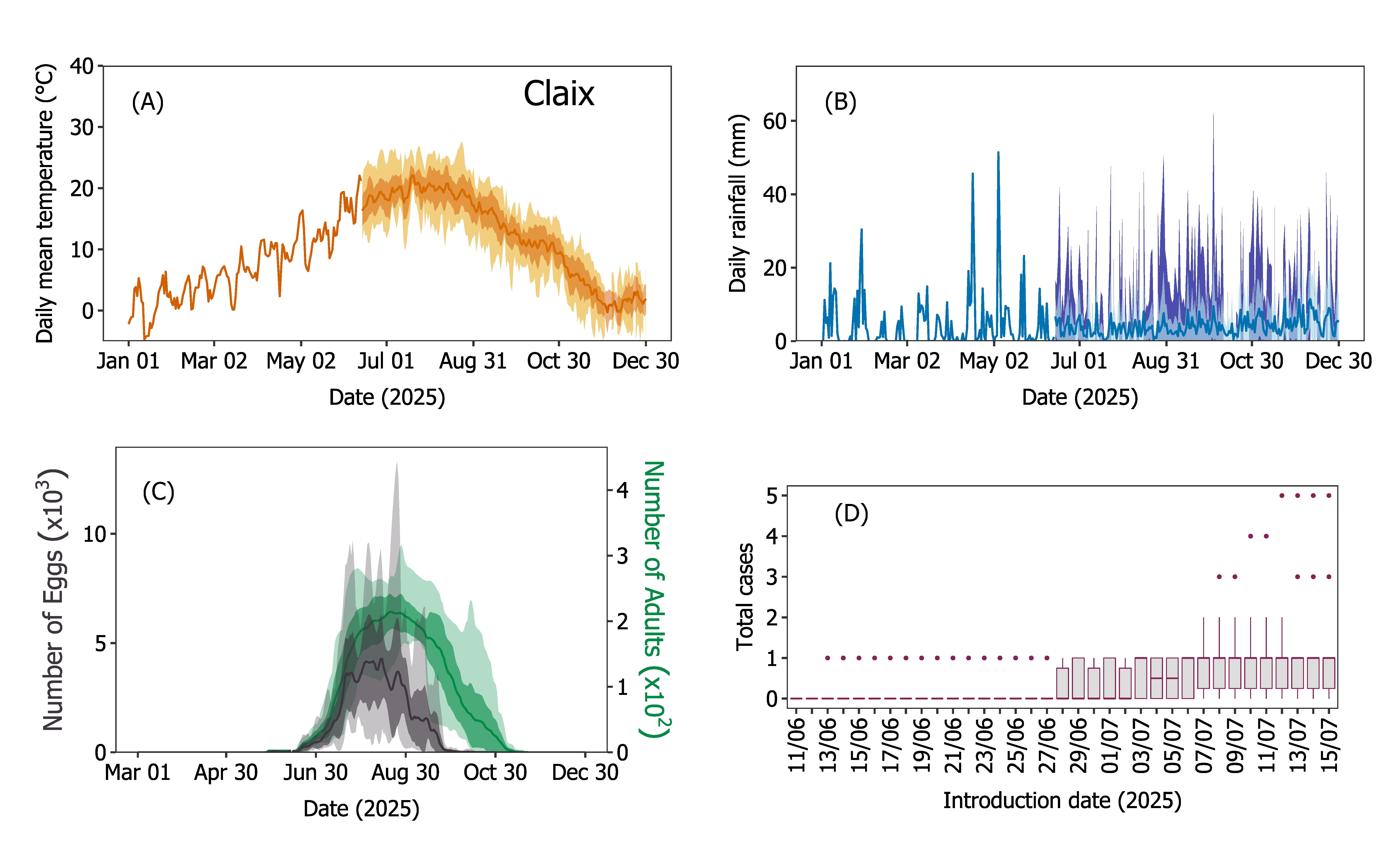


**Fig E.** Temperature, rainfall, adult *Ae. albopictus* dynamics with oviposition activity, and total predicted cases estimated for introduction dates spanning 3 weeks before to 2 weeks after the first reported symptomatic index case in Claix. Dots represent outliers.

**Fréjus**


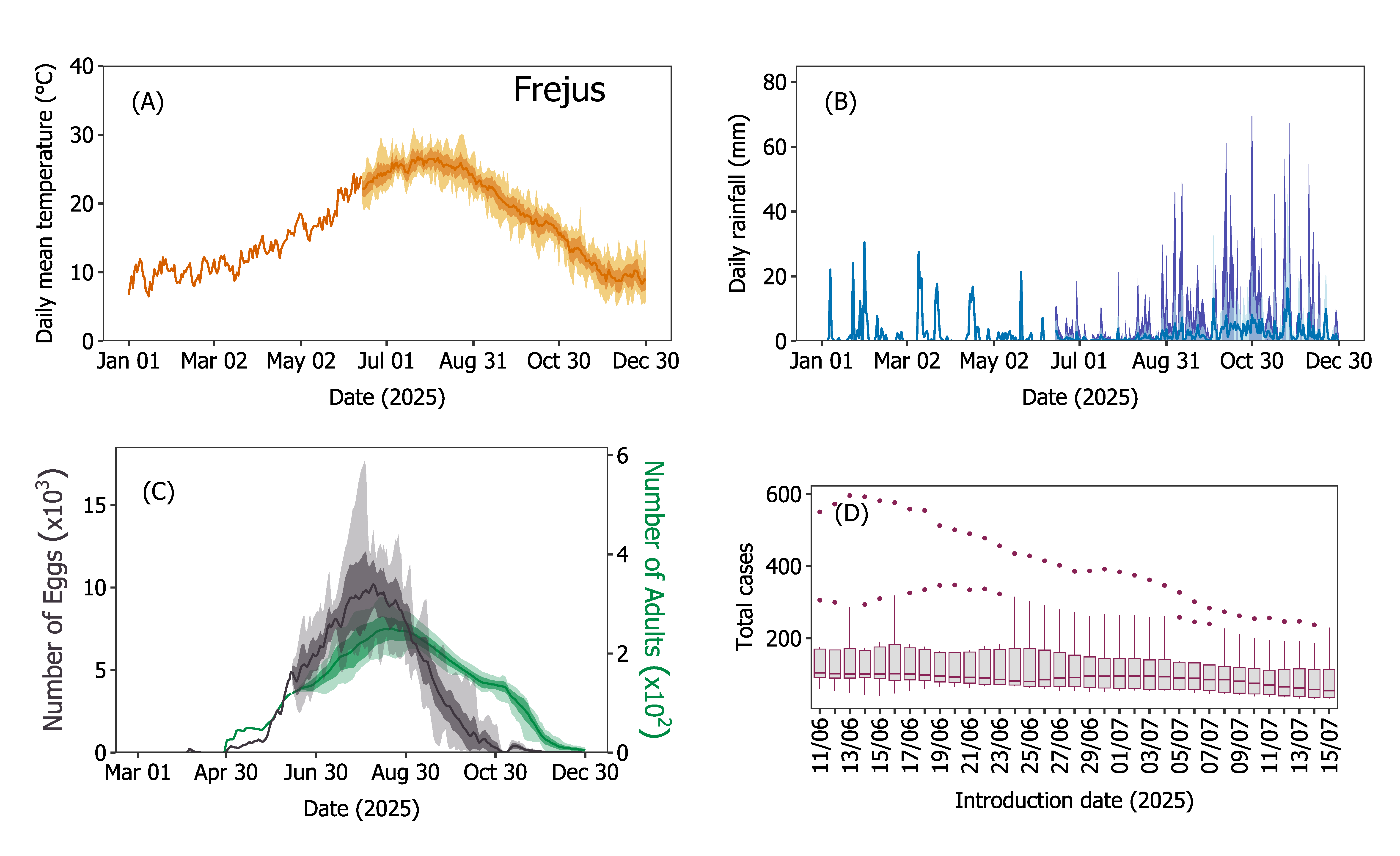


**Fig F.** Temperature, rainfall, adult *Ae. albopictus* dynamics with oviposition activity, and total predicted cases estimated for introduction dates spanning 3 weeks before to 2 weeks after the first reported symptomatic index case in Fréjus. Dots represent outliers.

**Grosseto-Prugna**





(D)

**Fig G.** Temperature, rainfall, adult *Ae. albopictus* dynamics with oviposition activity, and total predicted cases estimated for introduction dates spanning 3 weeks before to 2 weeks after the first reported symptomatic index case in Grosseto-Prugna.

**Grosseto-Prugna (inland)**


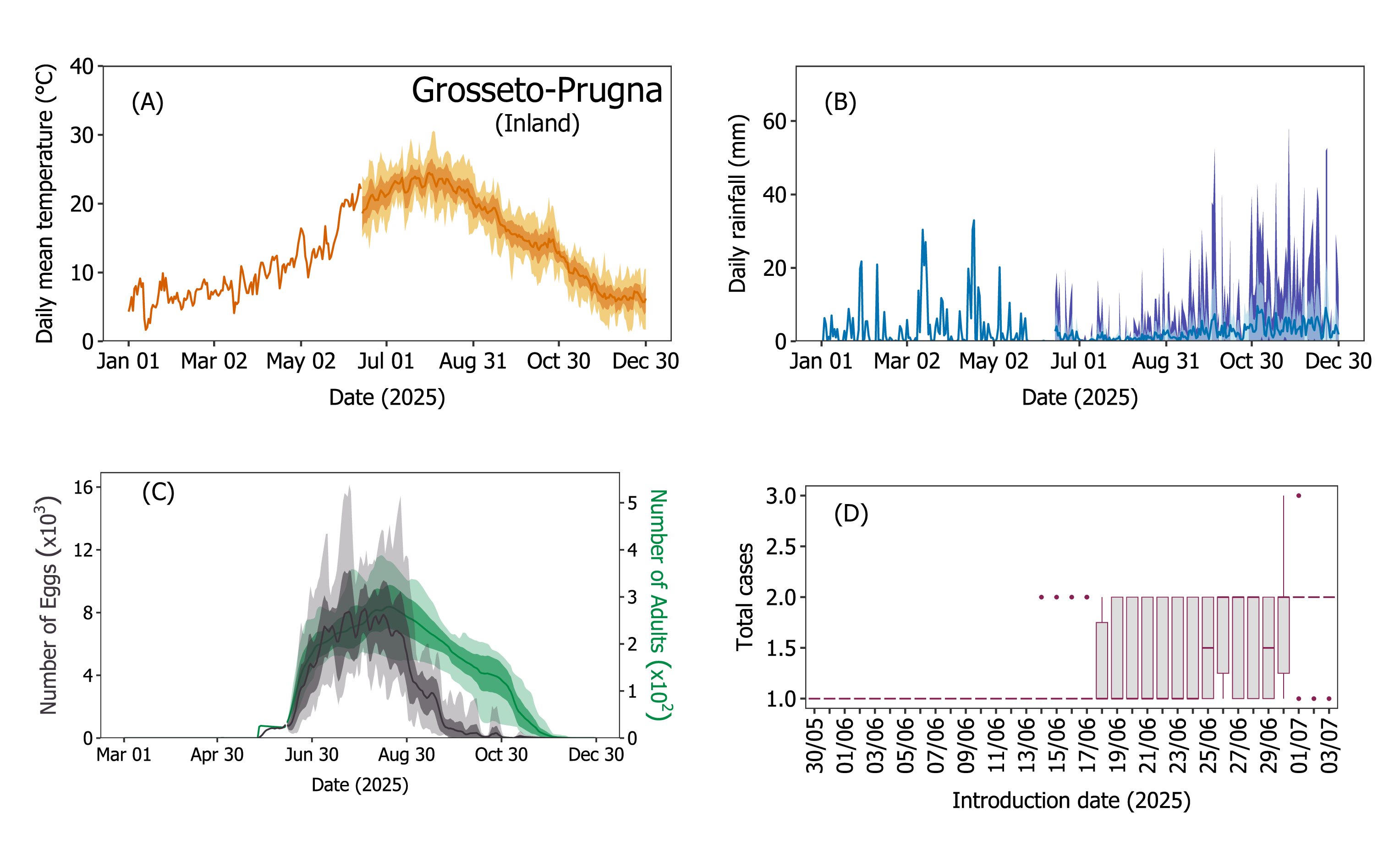


**Fig H.** Temperature, rainfall, adult *Ae. albopictus* dynamics with oviposition activity, and total predicted cases estimated for introduction dates spanning 3 weeks before to 2 weeks after the first reported symptomatic index case in Grosseto-Prugna (inland). Dots represent outliers.

**La Crau**


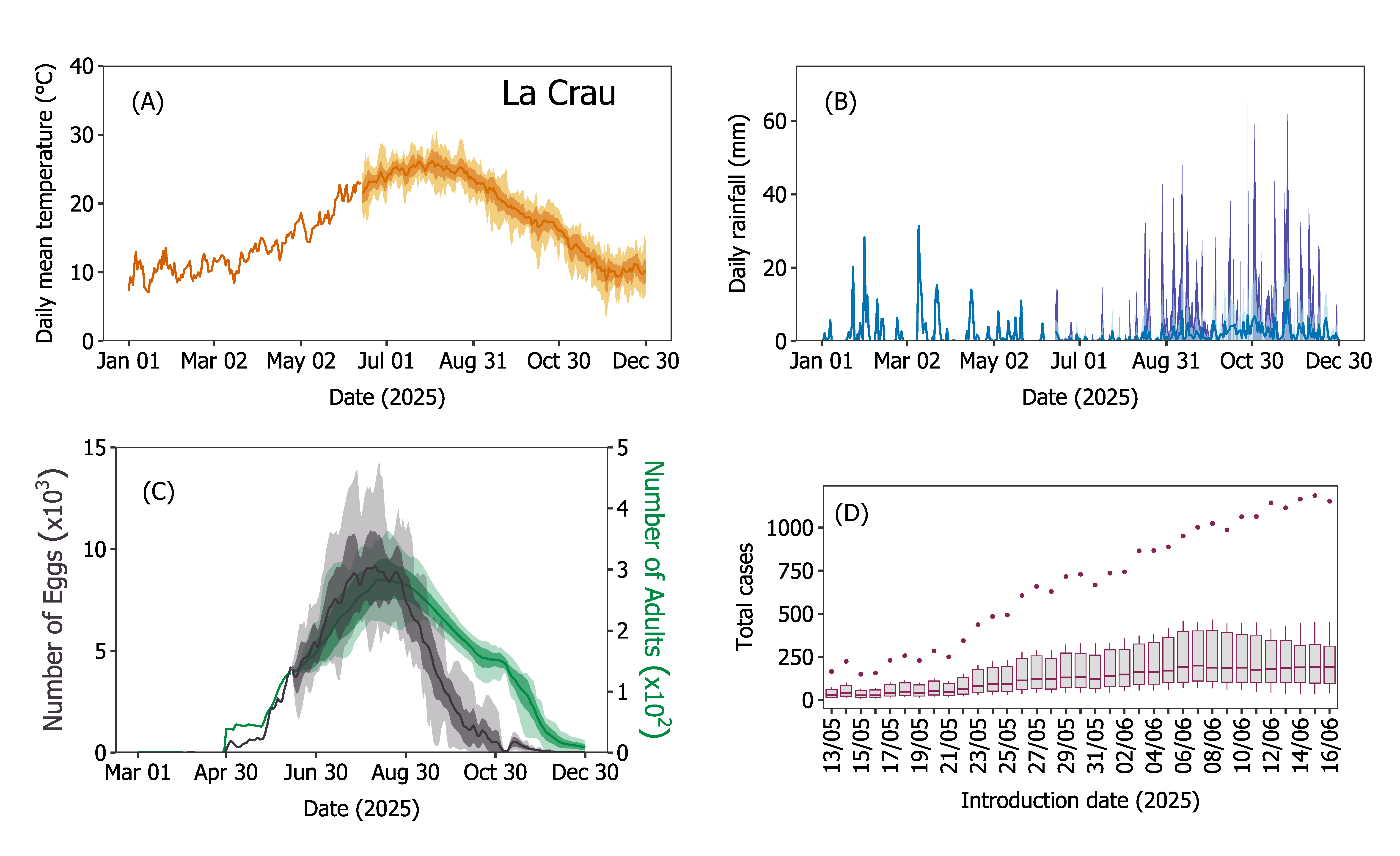


**Fig I.** Temperature, rainfall, adult *Ae. albopictus* dynamics with oviposition activity, and total predicted cases estimated for introduction dates spanning 3 weeks before to 2 weeks after the first reported symptomatic index case in La Crau. Dots represent outliers.

**Lipsheim**


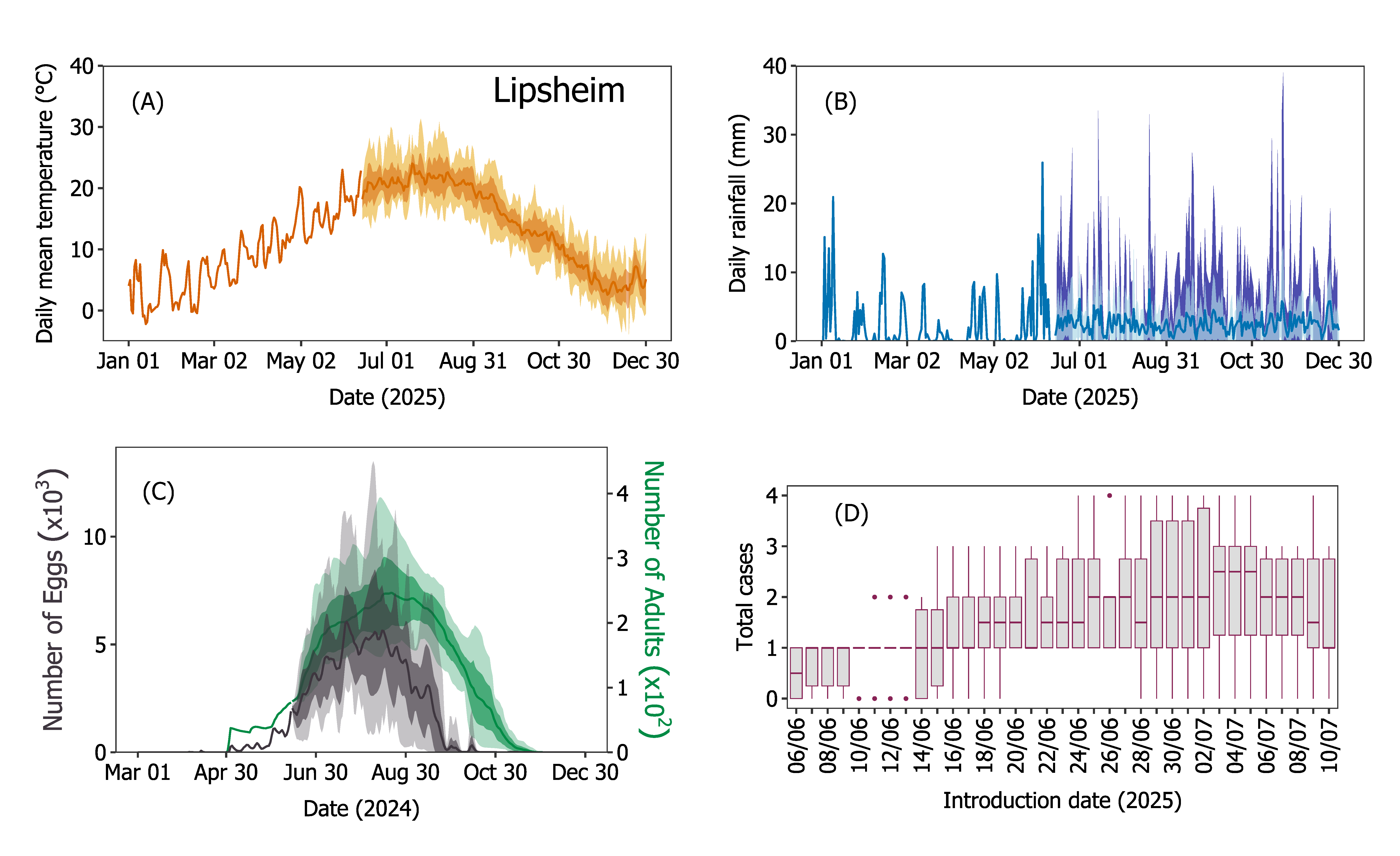


**Fig J.** Temperature, rainfall, adult *Ae. albopictus* dynamics with oviposition activity, and total predicted cases estimated for introduction dates spanning 3 weeks before to 2 weeks after the first reported symptomatic index case in Lipsheim. Dots represent outliers.

**Montoison**


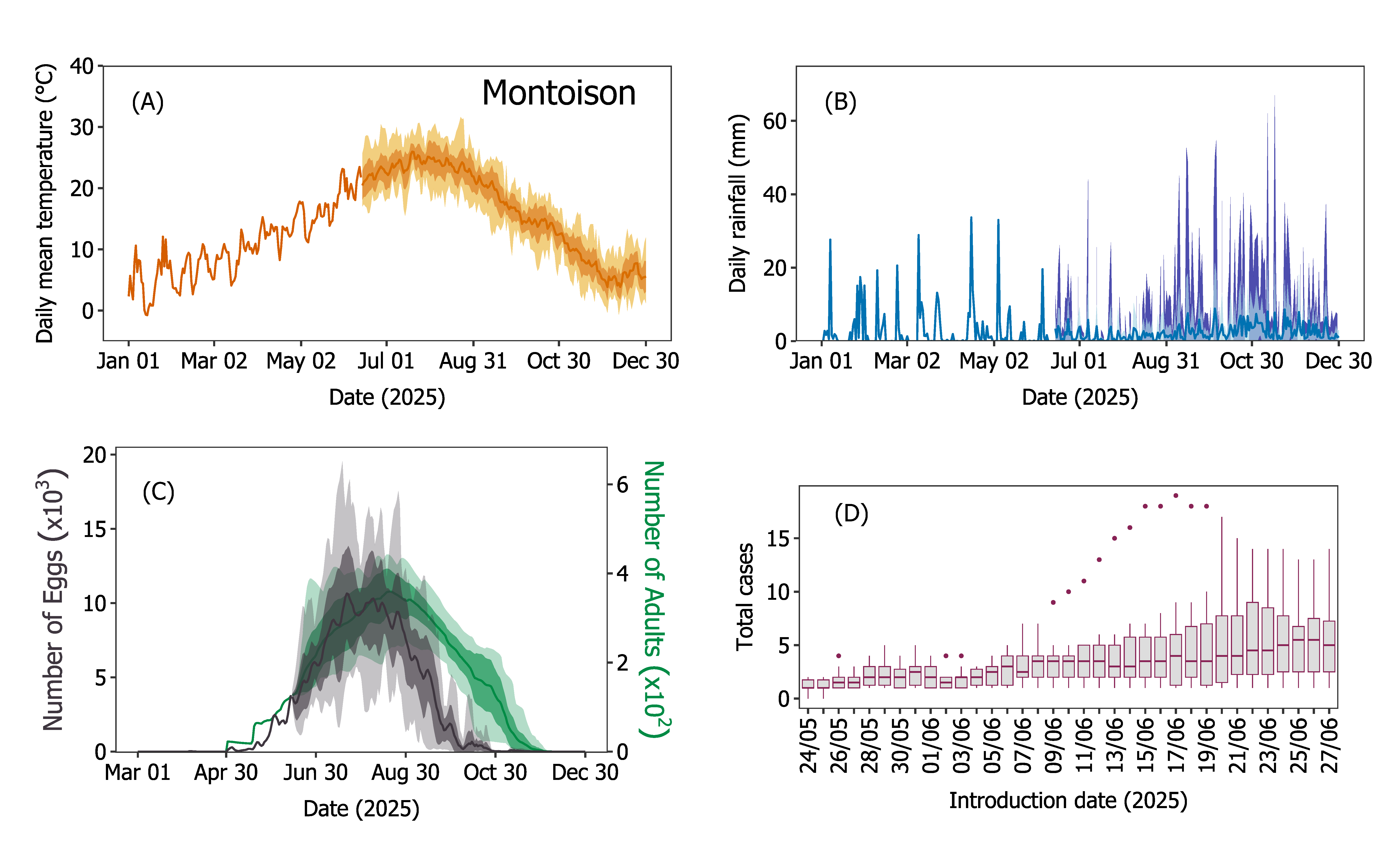


**Fig K.** Temperature, rainfall, adult *Ae. albopictus* dynamics with oviposition activity, and total predicted cases estimated for introduction dates spanning 3 weeks before to 2 weeks after the first reported symptomatic index case in Montoison. Dots represent outliers.

**Prades-le-Lez**


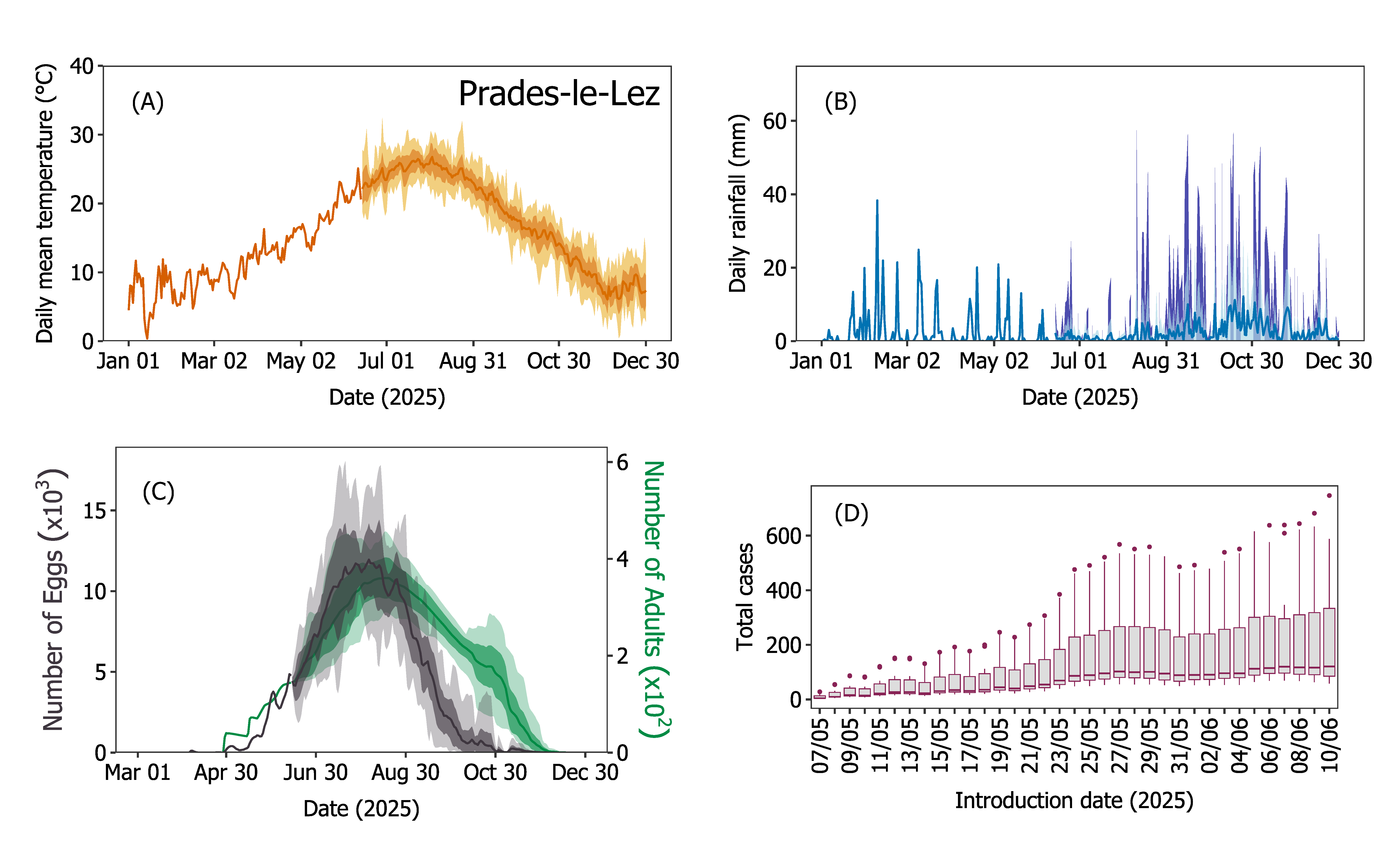


**Fig L.** Temperature, rainfall, adult *Ae. albopictus* dynamics with oviposition activity, and total predicted cases estimated for introduction dates spanning 3 weeks before to 2 weeks after the first reported symptomatic index case in Prades-le-Lez. Dots represent outliers.

**Salon-de-Provence**


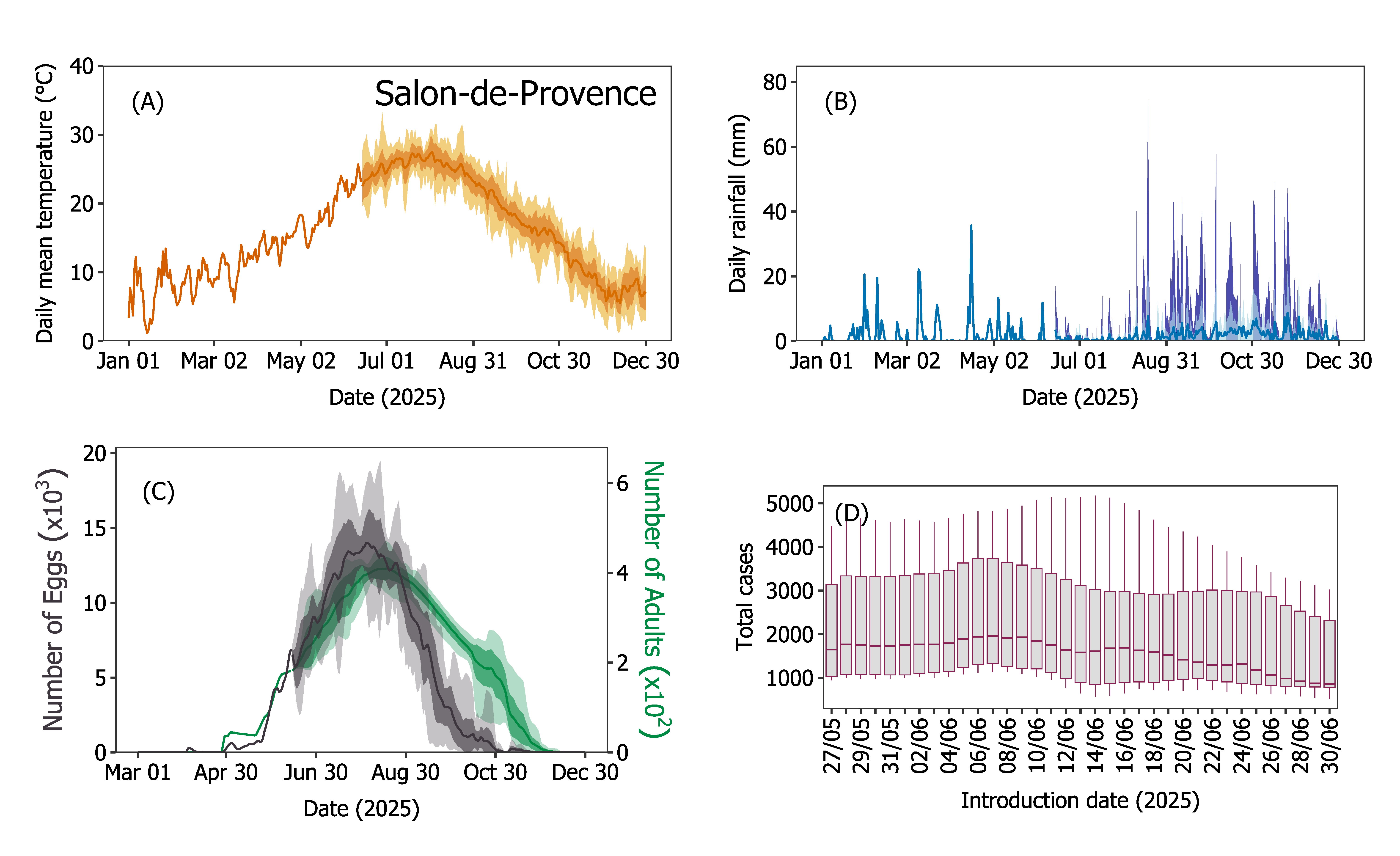


**Fig M.** Temperature, rainfall, adult *Ae. albopictus* dynamics with oviposition activity, and total predicted cases estimated for introduction dates spanning 3 weeks before to 2 weeks after the first reported symptomatic index case in Salon-de-Provence.

**Saint Bres**


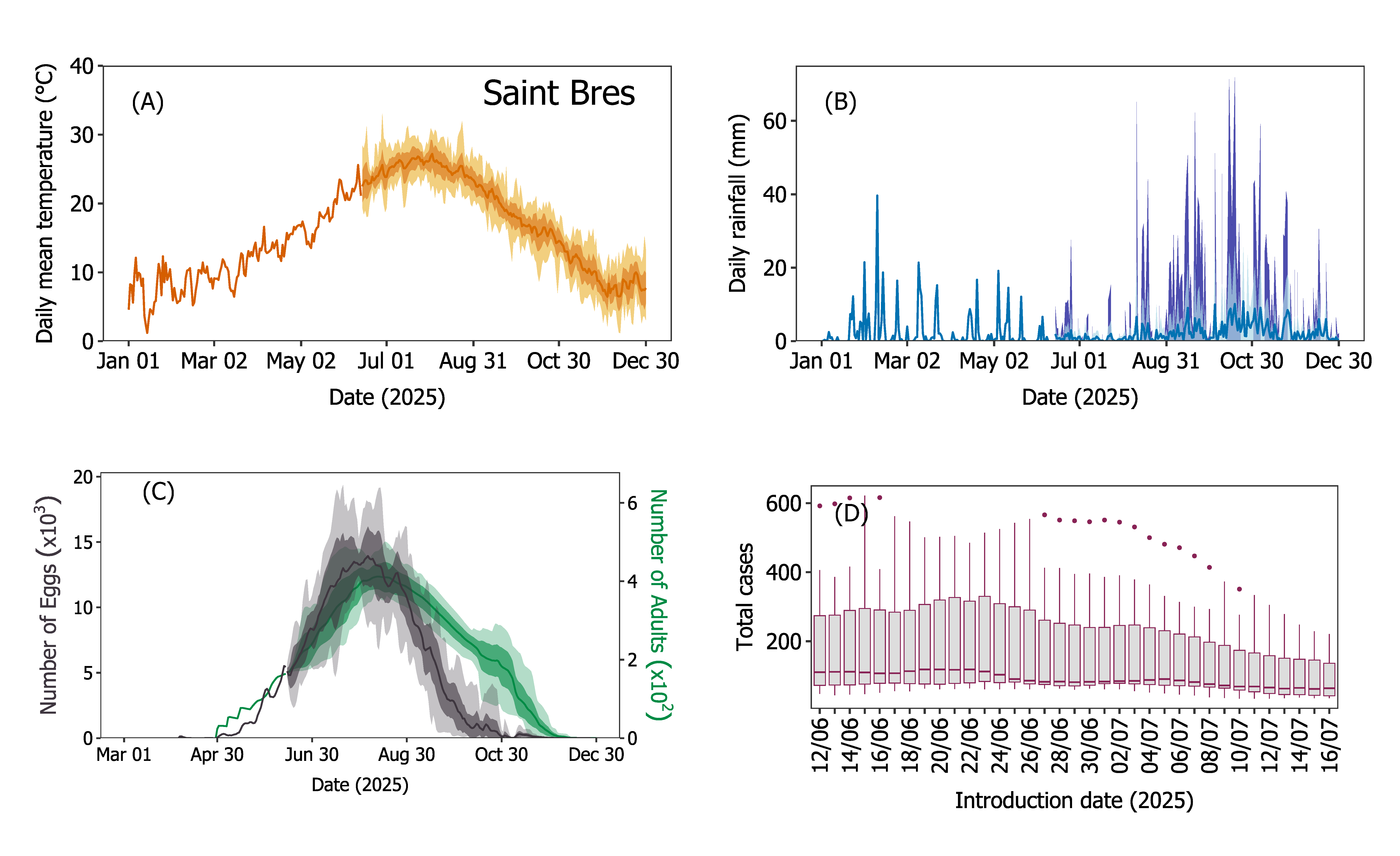


**Fig N.** Temperature, rainfall, adult *Ae. albopictus* dynamics with oviposition activity, and total predicted cases estimated for introduction dates spanning 3 weeks before to 2 weeks after the first reported symptomatic index case in Saint Bres. Dots represent outliers.

**Toulon**


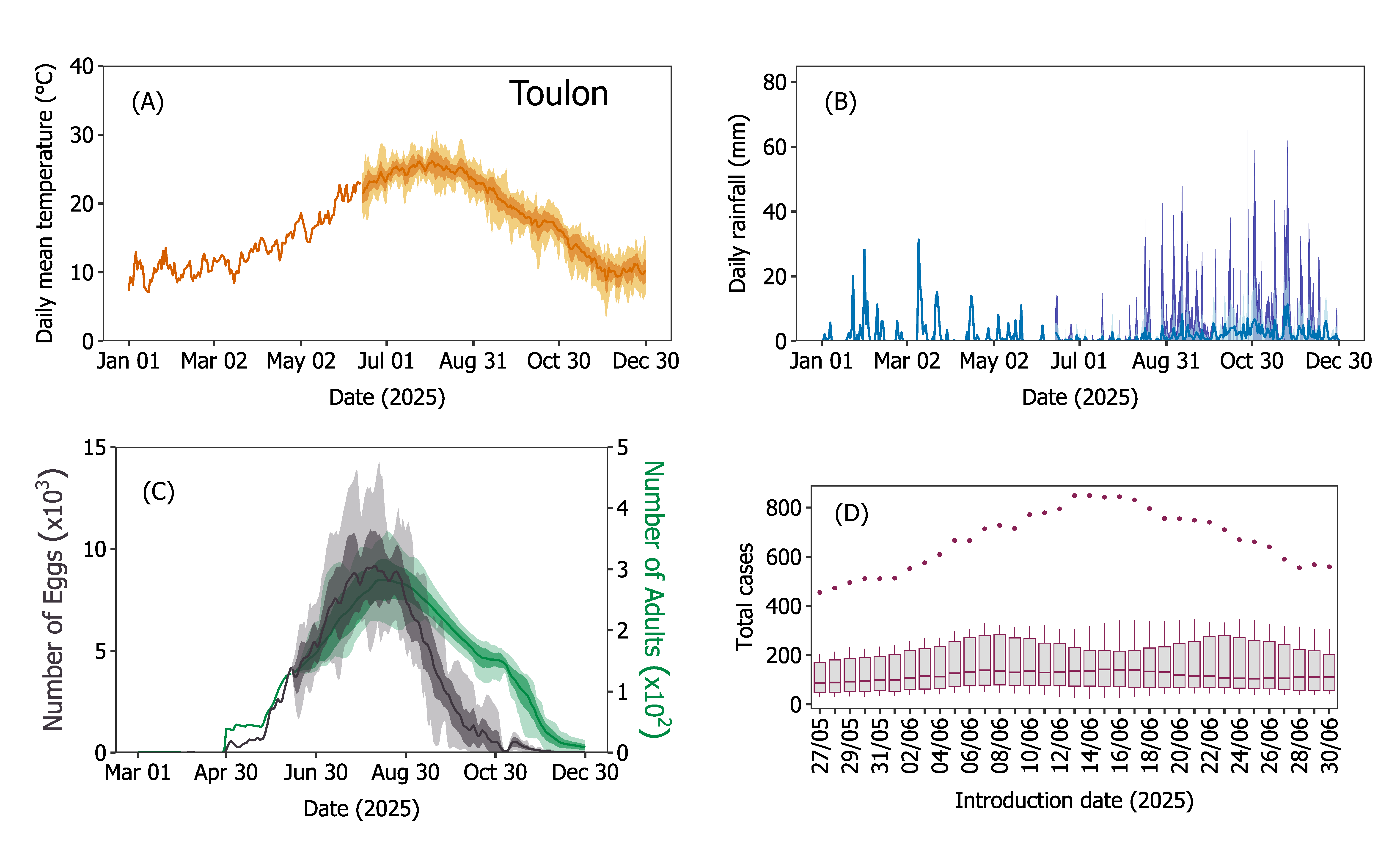


**Fig O.** Temperature, rainfall, adult *Ae. albopictus* dynamics with oviposition activity, and total predicted cases estimated for introduction dates spanning 3 weeks before to 2 weeks after the first reported symptomatic index case in Toulon. Dots represent outliers.

**References**

1. Mordecai, E.A., et al., *Detecting the impact of temperature on transmission of Zika, dengue, and chikungunya using mechanistic models.* PLOS Neglected Tropical Diseases, 2017. **11**(4): p. e0005568.

2. Rudolph, K.E., et al., *Incubation periods of mosquito-borne viral infections: a systematic review.* Am. J. Trop. Med. Hyg., 2014. **90**(5): p. 882–891.

3. Weaver Scott, C. and M. Lecuit, *Chikungunya Virus and the Global Spread of a Mosquito-Borne Disease.* New England Journal of Medicine. **372**(13): p. 1231–1239.
